# Hen Egg-Derived Bioactive Proteins and Peptides at the Gut Interface: From Molecular Characterization and Digestion to Microbiome Interactions and Prospective Precision Nutrition

**DOI:** 10.3390/foods15213849

**Published:** 2026-10-28

**Authors:** Osama K. Abou-Emera, Mohammed Aladhadh

**Affiliations:** 1 Department of Animal and Poultry Production, College of Agriculture and Food, Qassim University, Buraydah 51452, Saudi Arabia; 2 Department of Poultry Breeding Research, Animal Production Research Institute, Agriculture Research Centre, Dokki, Giza 12618, Egypt; 3 Department of Food Science and Human Nutrition, College of Agriculture and Food, Qassim University, Buraydah 51452, Saudi Arabia; aladhadh@qu.edu.sa

**Keywords:** hen egg (*Gallus gallus domesticus*), egg bioactive peptides, ovotransferrin, gut microbiota, intestinal barrier function, bioaccessibility, gastrointestinal digestion, prospective precision nutrition, evidence matrix

## Abstract

Hen eggs (*Gallus gallus domesticus*) contain bioactive proteins, peptides and lipids, but compound characterization, mechanistic cell and animal studies, and human consumption trials have developed largely in isolation. This narrative review, supported by a documented literature search, follows one pathway: egg composition, molecular characterization, processing and digestion, bioaccessibility, intestinal and microbial interaction, host signaling, and a prospective precision-nutrition outlook. Findings are classified into translational evidence categories (a heuristic classification of experimental systems, not a ranking of study quality) and summarized in a multidimensional evidence matrix. Egg white and yolk proteins, including ovotransferrin, ovomucin and lysozyme, yield characterized peptides, notably the ovotransferrin tripeptides IRW (Ile-Arg-Trp), IQW (Ile-Gln-Trp) and LKP (Leu-Lys-Pro), and ovomucin hydrolysates, whose activities in cell and animal models are compound-specific: LKP, for example, did not reproduce the effect of IRW on insulin resistance in muscle cells. In humans, one small ileostomy study (n = 5) reports higher ileal protein digestibility for cooked than raw egg, short-term trials show that whole-egg intake raises plasma choline, and an antibiotic-suppression study shows that trimethylamine-N-oxide formation after eggs plus phosphatidylcholine depends on gut microbiota. No study identified shows that IRW, IQW or LKP are generated, survive digestion or reach the circulation after ordinary egg consumption, and only six human studies address egg intake and the gut microbiome. Dose–response, delivery-system and safety data are largely missing, and precision nutrition for egg bioactives remains a research framework. Priorities are human pharmacokinetic confirmation, longer trials with shotgun metagenomics and functional readouts, and independent safety testing.

## 1. Introduction

Few single foods deliver as much nutritional value for so little cost as the hen’s egg (*Gallus gallus domesticus*). A whole egg supplies roughly 12–13 g of protein and 8–9 g of lipid per 100 g, alongside eighteen vitamins and minerals, for about 130 kcal per 100 g [1]. Egg proteins such as ovalbumin, ovotransferrin and lysozyme, the peptides released from them during processing and digestion, and the phospholipids and carotenoids concentrated in the yolk display antioxidant, antimicrobial, antihypertensive, anti-inflammatory and immunomodulatory activity under experimental conditions [1,2,3,4]. This dual identity, a complete food and a source of physiologically active molecules, makes the egg a natural test case for diet-derived bioactives. This review is restricted to eggs of the domestic chicken (*Gallus gallus domesticus*); eggs of other avian species are not considered.

Two shifts in how nutrition science evaluates such candidates have changed what counts as convincing evidence. First, activity in a test tube says little about relevance to someone who eats the compound: a molecule must survive, or be usefully transformed by, oral, gastric and intestinal digestion, and only the portion that remains accessible at the point of absorption can plausibly act on the host, so bioaccessibility and bioavailability separate a laboratory result from a nutritional one. Second, the gut microbiome is an active mediator between diet and host health: dietary components are metabolized by it, sometimes becoming active only after microbial transformation, and its composition responds in turn to diet [5]. Food bioactives shift the abundance of major phyla and taxa such as *Akkermansia muciniphila* and alter microbial metabolites, including short-chain fatty acids (SCFAs) and secondary bile acids, that signal to the epithelium [6]. Any claim that a food-derived bioactive improves host health therefore has to clear this intermediate layer.

A third development is interindividual variability: people respond differently to the same dietary exposure, partly through differences in gut microbial composition and function and in genetic, epigenetic and metabolic phenotype [7]. This has motivated precision nutrition, which aims to condition recommendations on an individual’s biological profile. The idea is well motivated but currently outruns the evidence: authoritative assessments conclude that, for generally healthy people, microbiome data cannot yet be translated into precise dietary recommendations without corroborating physiological biomarkers [8], and much interindividual variability in diet response remains unexplained [7,9]. Precision nutrition is therefore treated here as a research framework, not a validated practice.

Egg-derived bioactives sit at the intersection of all three developments, yet the literature on them has developed along separate tracks. One body of work characterizes egg proteins and their peptides, cataloging in vitro activities with little attention to digestive survival [3,4]. A second examines links between egg white protein or its peptides and intestinal barrier integrity or gut microbial composition, almost always in cell or animal models [10]. A third addresses whole-egg consumption and human gastrointestinal outcomes: a 2025 systematic review and meta-analysis found that egg intake was consistently associated with higher plasma choline, but that evidence for effects on the gut microbiome, inflammatory markers and trimethylamine-N-oxide (TMAO) was sparse and inconsistent, drawn mostly from short interventions and observational designs, and that essentially no human study isolates the egg-derived molecular species that might explain an effect of the whole food [11]. None of the three tracks alone connects molecular characterization through digestion and bioaccessibility to microbiome interaction and the individual-variability question that precision nutrition raises.

This review addresses that gap by tracing a single evidentiary pathway, from egg composition and the molecular characterization of its bioactive constituents, through processing and gastrointestinal digestion, to bioaccessibility, interaction with the intestinal epithelium and resident microbiota, and host immune and metabolic signaling. It then asks what this pathway does and does not yet imply for functional-food design and for precision nutrition. The specific questions are: (i) which egg-derived compounds are molecularly characterized, and by what means; (ii) which peptides and other bioactives are demonstrably released and stable during simulated and physiological digestion; (iii) what direct or microbiota-mediated evidence links them to intestinal barrier function and immune signaling; (iv) which reported microbiome effects reflect direct bioactive–microbe interaction rather than altered substrate availability; and (v) how far current evidence supports egg-bioactive-informed individualized recommendations rather than only a research agenda. The review emphasizes egg-white-derived proteins and peptides, for which mechanistic evidence is deepest, and treats yolk lipids mainly as a substrate for the choline–microbiota–trimethylamine-N-oxide (TMAO) pathway. Precision nutrition, safety and delivery are included only insofar as they bear on this central question. The review extends the evidentiary approach recently applied to whole-egg clinical research [11] to the molecular and mechanistic level.

### 1.1. Literature Search and Evidence-Synthesis Methods

This article is a narrative review supported by a structured, documented literature search. It is not a systematic review, and no review protocol was registered. It addresses hen (*Gallus gallus domesticus*) egg proteins, peptides, lipids and other components in relation to gastrointestinal digestion, intestinal epithelial function and the gut microbiome. Eggs of other avian species, egg-based delivery vehicles for non-egg compounds, and non-gastrointestinal outcomes (for example, joint health) were outside the primary scope and are mentioned only where they clarify the central pathway.

Searches were run in PubMed/MEDLINE and Europe PMC from database inception to 13 October 2026, limited to English-language articles. Scopus and Web of Science were not available to the authors and were not searched, which is a limitation of this review. Search strings combined egg terms (egg, “egg white”, “egg yolk”, ovotransferrin, ovomucin, lysozyme, phosvitin, livetin, “eggshell membrane”) with concept blocks for peptides and bioactivity (“bioactive peptide”, *hydrolysate*, IRW, IQW, LKP), digestion (“in vitro digestion”, INFOGEST, bioaccessib, *bioavailab*), the intestine (“intestinal barrier”, Caco-2, “tight junction”), the microbiome (“gut microbio”, metagenom, 16S, TMAO, choline) and precision nutrition (“precision nutrition”, “personalized nutrition”, interindividual variability), searched in titles and abstracts. In PubMed each concept block was combined with the egg terms in a separate search (596, 812, 554, 2046 and 29 records, respectively; 4037 before removal of duplicates); in Europe PMC a single combined search retrieved 3538 records. The strings and counts are given in Supplementary Table S1.

Study selection proceeded in three steps. First, records from both databases were combined, and duplicates were identified by matching titles and first-author surnames and removed manually (no systematic-review software was used, consistent with this narrative review design). Second, title and abstract screening was applied: records were retained if they concerned an egg-derived protein, peptide, lipid or metabolite in relation to gastrointestinal digestion or stability, intestinal epithelial function, gut microbial composition or activity, or host gastrointestinal or metabolic outcome. Third, records passing screening were retrieved in full text and assessed against inclusion criteria: English-language primary research articles and systematic reviews examining an egg-derived compound in the defined scope. Excluded were conference abstracts; commentaries without primary data; studies treating non-hen avian eggs as primary subject; studies using egg components exclusively as delivery vehicles for non-egg bioactives; and non-gastrointestinal outcomes. Systematic and narrative reviews were included as secondary sources to locate primary studies and not assigned their own evidence category. Study quality was assessed qualitatively for each major finding; no formal risk-of-bias instrument was applied.

Searching only PubMed/MEDLINE and Europe PMC is a limitation acknowledged throughout. Scopus and Web of Science were not available and were not searched; relevant studies indexed only in those databases may not have been retrieved. All statements that “no study was identified” refer to this two-database search and should not be read as confirmed global absence of evidence.

Primary research articles were prioritized for every claim about the presence or absence of evidence; systematic and narrative reviews were used to frame context and to locate primary studies and are cited as secondary sources. Studies were included if they examined an egg-derived protein, peptide, lipid or metabolite in relation to digestion, absorption, intestinal epithelial function, the microbiota or host outcomes, or if they reported human data on whole-egg intake and gastrointestinal outcomes. Data were extracted narratively. The six human studies of egg intake and gut microbiome composition identified by a recent systematic review [11] were consulted in their original reports [12,13,14,15,16,17] and are summarized in Section 6.1.

Each finding was assigned to one or more translational evidence categories according to the experimental system used: Category 1, in silico; 2, chemical or biochemical assay; 3, in vitro cellular study; 4, simulated gastrointestinal digestion; 5, animal study; 6, human observational study; 7, human intervention or clinical studies. The categories describe which question a study can answer and where it sits between the molecule and a human outcome. They are a heuristic framework, not a validated hierarchy, and not an ordinal ranking of study quality or strength of inference. Because they address different questions, findings are summarized across eight evidence dimensions in a multidimensional evidence matrix (Section 11.1). Study quality (design, sample size, confounding, dosing) was appraised qualitatively and is reported in the text and tables; no formal risk-of-bias scoring was applied. Statements that “no study was identified” mean that none was retrieved by the search described above; they are not claims of exhaustive proof of absence.

### 1.2. Conceptual Framework

The review is organized along one pathway: egg composition → processing → gastrointestinal digestion → bioaccessibility and absorption → intestinal epithelium and microbiota → host immune and metabolic signaling → health outcome (shown in full in Section 14). Each link is labeled [E] established, [P] probable mechanism or [H] proposed hypothesis. [E] means supported by direct evidence for egg-derived compounds at the level claimed; [P] means supported by strong indirect or analogous evidence but not demonstrated for the specific compound, food form or exposure; [H] means mechanistically plausible but untested for egg-derived bioactives. General physiological evidence extrapolated from non-egg contexts is never labeled [E] for egg. Section 2, Section 3, Section 4, Section 5, Section 6, Section 7, Section 8, Section 9 and Section 10 are organized around these links, and Section 14 revisits which links are supported and which are missing.

## 2. Molecular Landscape of Egg-Derived Bioactives

### 2.1. Egg Proteins

Egg white is roughly 90% water and 10% protein, and that protein fraction is dominated by a small number of well-characterized species. Ovalbumin accounts for about 54% of total egg white protein; ovomucoid and ovotransferrin each contribute close to 10%; ovomucin and lysozyme each make up around 3%; and the remainder is distributed among minor proteins—ovoinhibitor, ovoglycoprotein, ovoflavoprotein, ovomacroglobulin, avidin, and cystatin [1]. Ovalbumin’s biological role is not firmly established; it is generally regarded as a storage protein and amino acid source rather than a dedicated defense molecule [1]. Ovomucoid, by contrast, is the dominant food allergen in egg white [1], a property directly relevant to any downstream functional-food application and revisited in Section 10 of this review.

Of the egg white proteins, ovotransferrin is the most thoroughly characterized from a gut-relevant standpoint. It is an iron-binding glycoprotein whose two homologous lobes each chelate one Fe^3+^ ion; this iron restriction is considered the principal basis of egg white’s resistance to pathogens such as *Salmonella* Enteritidis [18]. A 2021 mechanistic review concluded that ovotransferrin also has an iron-independent antibacterial activity involving perturbation of the bacterial membrane, but cautioned that most of the evidence comes from purified-protein assays outside the alkaline, viscous environment of intact egg white, and that translation to that setting, let alone the gastrointestinal lumen, is only partly resolved [18]. Ovomucin, the glycoprotein responsible for the gel-like consistency of thick egg white, and lysozyme, a small glycoside hydrolase, complete the major antimicrobial-associated proteins [1]. Lysozyme cleaves the β-1,4 glycosidic bond between N-acetylmuramic acid and N-acetylglucosamine in bacterial peptidoglycan, acting mainly against Gram-positive bacteria [19]. Orally administered hen egg white lysozyme (HEWL) also interacts with gut-associated lymphoid tissue (GALT) and intestinal lymphocytes in a manner that a 2023 pharmacological review concluded is not fully dependent on its enzymatic activity, making lysozyme one of the few egg proteins with a documented, if incompletely mapped, route of action at the intestinal interface [19].

### 2.2. Bioactive Peptides

The clearest example of a well-characterized egg-derived bioactive peptide family comes from enzymatic hydrolysis of ovotransferrin, which yields the tripeptides IRW (Ile-Arg-Trp, 473.6 Da), IQW (Ile-Gln-Trp, 445.5 Da), and LKP (Leu-Lys-Pro, 356.5 Da) [20]. All three were first identified as angiotensin-converting enzyme (ACE) inhibitors in vitro, but their sequence–activity relationship diverges further downstream. IRW and IQW also suppress markers of oxidative stress and pro-inflammatory signaling in cultured human umbilical vein endothelial cells [21], and administering IRW to spontaneously hypertensive rats (SHR) not only lowers blood pressure but upregulates angiotensin-converting enzyme 2 (ACE2) and activity along the ACE2/angiotensin-(1–7)/Mas receptor axis in the mesenteric artery—a mechanism distinct from simple ACE inhibition [20,22]. Of the same three tripeptides, only IRW significantly improved angiotensin-II-stimulated insulin resistance in L6 rat skeletal muscle cells, accompanied by reduced reactive oxygen species formation, whereas IQW and LKP did not improve insulin resistance [23]. This peptide family illustrates a point developed in Section 11: the depth of mechanistic characterization—moving from an in vitro enzyme assay to endothelial and myotube cell culture to a whole-animal SHR model—is genuinely unusual for a food-derived peptide, yet every study underpinning it remains a cellular or rodent experiment. No study retrieved for this review has shown that IRW, IQW or LKP are generated in comparable quantities during human digestion of whole eggs, survive intestinal transit intact, or reach systemic circulation at concentrations approaching those administered experimentally (Section 4.4, Section 4.5 and Section 4.6). Throughout this review, a peptide is therefore described either as *experimentally generated* (formed from a purified protein or hydrolysate under defined enzymatic conditions, with or without reported stability to further simulated digestion) or as *demonstrated* (shown to be produced during digestion of whole cooked egg, to survive transit and to reach the circulation after normal consumption). For IRW, IQW and LKP only the first status is documented in the sources reviewed here. Beyond the ovotransferrin tripeptides, egg white proteins and their hydrolysates as a class have been reported to yield further peptides with ACE-inhibitory, antioxidant, antimicrobial, anti-inflammatory, antidiabetic, and mineral-binding activity across a range of parent proteins and digestion or fermentation conditions [3,4], though the rigor of structural confirmation and functional validation varies considerably across this literature (Section 3). Table 1 lists the peptides discussed, with parent protein, sequence, activity, experimental model and evidence category. Evidence categories follow Section 1.1: an assay on plasma or other material taken from untreated animals is classified as a biochemical assay (Category 2), Category 5 is assigned only where animals were dosed, and entries drawn from review articles are secondary sources that are not assigned a category. Cell models are Caco-2 (human colon-adenocarcinoma-derived intestinal epithelial cells) and IPEC-J2 (porcine small-intestinal epithelial cells); ETEC is enterotoxigenic *Escherichia coli*.

### 2.3. Egg Yolk-Derived Bioactives

Egg yolk holds nearly all of the egg’s lipid, about 8.7 g per 100 g of whole egg, as triglycerides and fatty acids (~62% of yolk lipid), phospholipids (~33%) and cholesterol (~5%) [1]. Phosphatidylcholine accounts for roughly 78.5% of yolk phospholipid and phosphatidylethanolamine for about 17.5% [30]; because phosphatidylcholine is also a principal dietary source of choline, this fraction links directly to the choline–gut microbiota–trimethylamine axis of Section 6, independent of any peptide-mediated mechanism. Yolk lipoproteins form an insoluble granular fraction (mostly high-density lipoprotein, with low-density lipoprotein and the iron-binding phosphoprotein phosvitin) and a soluble plasma fraction dominated by low-density lipoprotein and the livetins (α-livetin, serum albumin; β-livetin, a glycoprotein; γ-livetin, yolk immunoglobulin Y [IgY]) [1]. The livetin fraction and its hydrolysates suppress lipopolysaccharide-induced inflammatory signaling in cultured macrophages, but this remains in vitro evidence [31]. Phosvitin’s high phosphoserine content confers iron chelation and antioxidant activity, and its strong negative charge makes it comparatively resistant to proteolysis, which bears on how much intact or partially degraded phosvitin reaches the intestine [1].

### 2.4. Eggshell and Eggshell Membrane Components

The eggshell membrane (ESM) is compositionally distinct from egg white and yolk, consisting principally of collagen-type proteins (including collagen types I, V, and X), sulfated glycosaminoglycans such as chondroitin sulfate, and hyaluronic acid [32]. In vitro simulated gastrointestinal digestion of natural eggshell membrane hydrolyzes over 40% of its protein and releases short antioxidant peptides—including the sequences TEW, SWVE, and VYL—confirming that ESM protein is at least partially digestible and that peptide fragments are, in principle, released in the gut lumen [32]. The documented downstream health effects of ESM and its hydrolysates, however, are overwhelmingly oriented toward joint and connective-tissue outcomes: chondrocyte protection against oxidative stress, and, in randomized human trials, improvement in osteoarthritis joint symptoms, rather than any gastrointestinal or microbiome endpoint [32]. Consistent with the scope boundary this review applies to eggshell-derived material, ESM is therefore included here only to note that a gastrointestinal digestion step relevant to the review’s central pathway has been directly demonstrated for it, while flagging that no evidence identified for this review links ESM or its peptides to intestinal barrier function, gut microbiome composition, or any other gut-interface endpoint.

### 2.5. Other Bioactive Molecules

Eggs additionally supply fat-soluble micronutrients—vitamins A, D, and E, and the yolk carotenoids lutein and zeaxanthin—together with B vitamins and trace minerals including selenium, zinc, and iron [1]. These compounds are nutritionally well established and, for several, carry documented in vitro antioxidant or immunomodulatory activity as part of the broader class of egg nutrients [1]. They are not treated here as a distinct gut-interface bioactive class: no evidence located for this review demonstrates a mechanism by which egg-derived, as opposed to supplement-derived or other dietary-source, vitamin A, D, E, or carotenoid intake specifically modulates gut microbial composition or intestinal barrier function. Where such a mechanism does exist for choline—via its yolk phospholipid source and downstream microbial metabolism—it is treated in Section 2.3 and developed further in Section 6, rather than duplicated here. Major egg-derived bioactive compounds, molecular origin, reported activities, and evidence category are shown in Table 2.

## 3. Molecular Characterization and Advanced Analytical Approaches

### 3.1. Proteomics and Peptidomics

Characterization of egg-derived bioactives rests almost entirely on mass-spectrometry workflows, and what matters for this review is how far each workflow carries a candidate toward biological relevance. A 2023 proteomic atlas combined nano-liquid chromatography–tandem mass spectrometry (nanoLC-MS/MS) analysis of 45 fresh eggs with a literature synthesis and cataloged 808 egg white and 813 yolk proteins, 229 of them shared [36]; a review of two decades of egg proteomics traces the progression from two-dimensional gel electrophoresis and peptide mass fingerprinting to shotgun LC-MS/MS and the annotation of low-abundance proteins [37]. These catalogs establish what is present in raw egg, not what is released, stable or absorbed after ingestion. At the peptide level, matrix-assisted laser desorption/ionization time-of-flight (MALDI-ToF) mass spectrometry with database-matched sequence identification (for example, Mascot) is the standard route to confirming the sequence of a peptide isolated by chromatographic fractionation, as shown for four antioxidant, ACE-inhibitory and antidiabetic peptides assigned to apolipoprotein B, vitellogenin-2 or apovitellenin-1 in a pepsin hydrolysate of defatted yolk protein [28]. Structural confirmation of this kind separates a peptide with an established sequence, mass and parent protein from one merely inferred to exist in a hydrolysate; it does not show that the peptide forms under physiological digestion (Section 4).

### 3.2. In Silico Prediction, Molecular Docking, and Their Limits

In silico approaches complement experimental fractionation: virtual proteolysis of a known sequence, scoring of the fragments with tools such as PeptideRanker or the BIOPEP-UWM database, prediction of toxicity and absorption, and molecular docking against a target such as dipeptidyl peptidase-4 (DPP4). A recent egg yolk study shows the approach at its most complete: virtual proteolysis with alcalase and pepsin, machine-learning and BIOPEP-based scoring and docking narrowed the candidates to five DPP4-inhibitory peptides, and the top candidate (AAQEKIRYW) was synthesized and confirmed in a cellular assay and in mice (oral glucose tolerance in healthy mice; plasma glucose and DPP4 activity in type 2 diabetic mice), with a defined half-maximal inhibitory concentration (IC_50_) and a docking pose supported by molecular dynamics [26]. That chain, prediction followed by wet-lab confirmation in cells and animals, is the standard against which prediction-only claims should be read: a docking score or PeptideRanker probability is a hypothesis about where experimental effort is likely to pay off, not evidence of bioactivity. A 2026 methodological review of artificial intelligence for food-derived peptides reached a similar conclusion for the field, citing the lack of standardized training databases and arguing that integration of metabolomic and microbiota data is a precondition, not yet an achievement, for personalized-nutrition applications [38].

### 3.3. Lipidomics and Metabolomics: An Underdeveloped Layer

Lipidomic and metabolomic characterization specific to egg-derived bioactives is markedly less developed than the protein and peptide literature. Compositional analysis of yolk phospholipid classes exists at the level described in Section 2.3 [30], but this review did not locate comparable mass-spectrometry-based lipidomic profiling tracking how individual phospholipid or sphingolipid species change across digestion, nor targeted metabolomic studies mapping the small-molecule products generated when egg lipids or proteins are metabolized by gut microbiota specifically, as distinct from the broader diet-microbiome metabolomics literature surveyed in Section 6. This is a genuine gap rather than a matter of scope: the digestion-and-microbiome pathway this review traces depends on knowing what molecular species actually persist and transform at each step, and for the lipid fraction of the egg, that evidence base is considerably thinner than for the protein fraction.

### 3.4. The Standing Distinction: Predicted Versus Demonstrated Bioactivity

**Figure 1:**
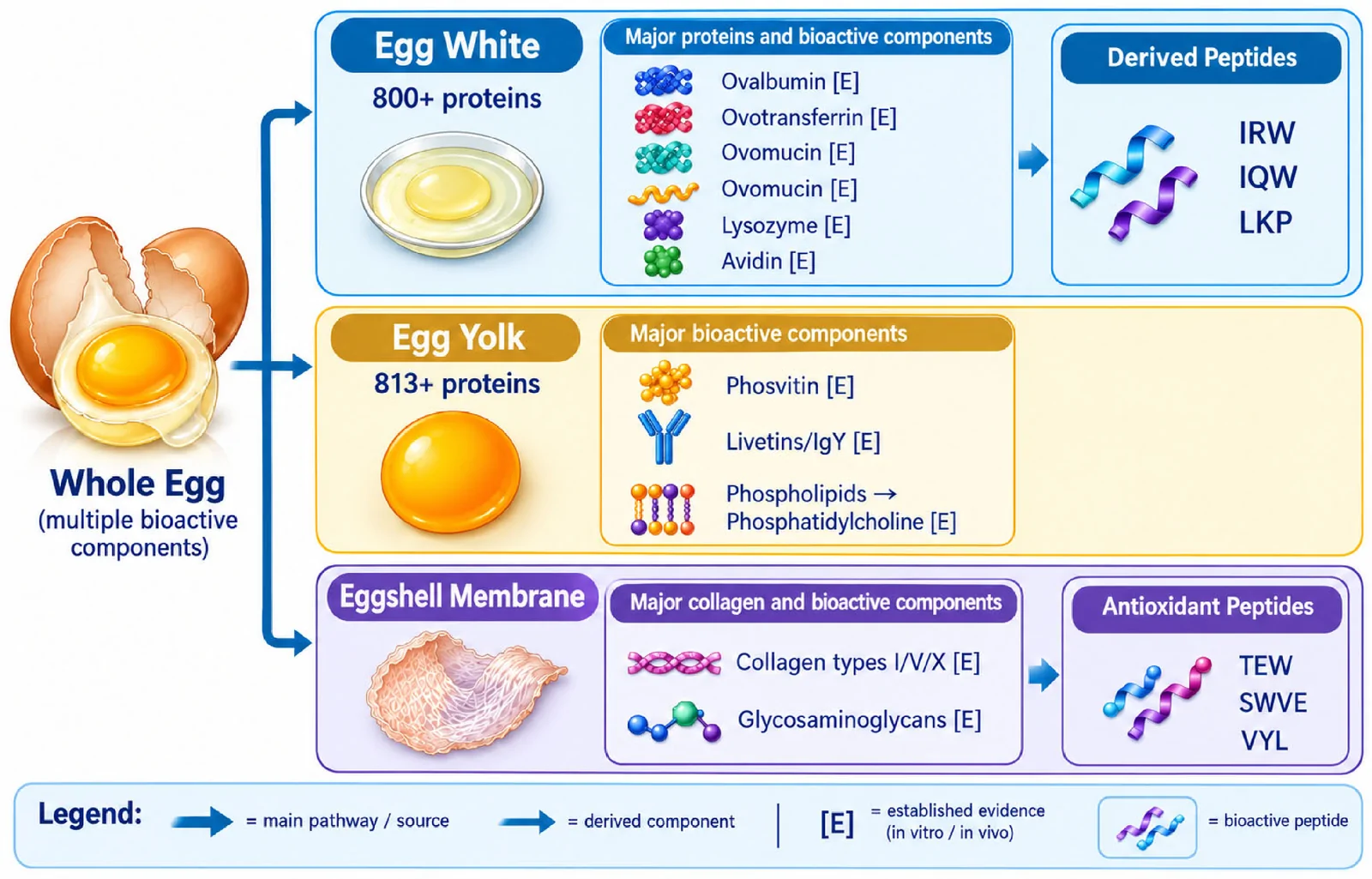
Molecular landscape of egg-derived bioactives. Egg proteins and peptides are described in Section 2 and Section 3; [E] indicates established evidence, as defined in Section 1.2. Figure generated with the assistance of ChatGPT (GPT-5.5; OpenAI, San Francisco, CA, USA); content reviewed, edited, and approved by the authors.

Across Section 2 and Section 3, a consistent asymmetry deserves to be stated plainly before the review turns to digestion. Egg-derived peptides with the deepest mechanistic characterization—the ovotransferrin tripeptides IRW, IQW, and LKP (Section 2.2)—reached that depth through years of sequential cell- and animal-level experimentation following their initial identification, not through computational prediction. Peptides identified primarily through recent in silico pipelines more often carry only a docking score and, at best, a single downstream cellular or short-term animal validation [26]. Neither route yet reaches human evidence for any egg-derived peptide considered in this review. The practical implication carried forward is that a peptide’s presence in a published in silico screen, an egg-protein-derived predicted-activity table, or even a BIOPEP database entry should not be read as experimentally confirmed bioactivity, and still less as evidence of activity after human digestion of the intact food (Section 4). Three conclusions follow from Section 2 and Section 3. First, molecular characterization is mature for the egg proteome but not for egg lipids or for post-digestion species. Second, the depth of functional characterization is highly uneven: a few peptides (IRW, IQW, LKP) have been studied from enzyme assay to animal model, whereas most listed sequences rest on a single assay or on prediction. Third, structural confirmation, prediction and functional validation are distinct steps, and a peptide’s position along them should be stated whenever it is cited. Figure 1 summarizes the molecular landscape of egg-derived bioactives.

## 4. Processing, Digestion, and Release of Bioactive Compounds

### 4.1. Processing Effects: The Clearest Evidence Concerns Heat

Of the processing variables relevant to egg bioactives (cooking, pasteurization, fermentation, enzymatic hydrolysis and high-pressure processing), the egg-specific, digestion-relevant evidence retrieved for this review is concentrated on thermal treatment. The most physiologically direct finding comes from a single small study: in five ileostomy patients given isotope-labeled egg protein, true ileal digestibility was 90.9% for cooked and 51.3% for raw egg protein, about 1.8-fold higher after cooking [39]. The difference is generally explained by heat-induced unfolding of the native protein and inactivation of protease inhibitors, which makes cleavage sites accessible to digestive proteases [11,39]. This is one study in five participants with an ileostomy, published in 1998; it should not be generalized to the wider population (for example, people with an intact colon, infants, older adults or patients), although it is one of the few measurements in this review obtained directly in living humans rather than by simulation, cell culture or animal model. Egg-white-specific heat-treatment studies extend the mechanism further: pepsin digestibility of egg white protein peaked at a 65 °C pretreatment, while combined pepsin-plus-pancreatin digestibility rose continuously with increasing cooking temperature up to 100 °C, and the resulting digests contained proportionally more low-molecular-weight peptides at higher pretreatment temperatures [40]. A separate study manipulating both pH and heat treatment of egg white powder before simulated digestion found that these two variables jointly determine the degree of protein aggregation and solubility, which in turn shapes how much antioxidant peptide activity is released downstream during digestion [41]. Cooking method matters at the whole-food level as well: comparing hard-boiled, poached, and omelet preparations under a simulated elderly gastrointestinal model, one study found that omelet preparation reduced proteolysis by up to 37% relative to a standard adult model, while hard-boiled and poached preparations were comparatively more digestible under the same altered conditions [42]—a finding this review returns to in Section 4.2, and again for its individual-variability implications in Section 8. By contrast, egg-specific, digestion-relevant evidence for fermentation or high-pressure processing is comparatively thin. Where these technologies appear elsewhere in the egg-processing literature, they are more often evaluated for shelf-life, texture, or general functional-property outcomes than for their downstream effect on gastrointestinal digestion or bioactive-peptide release, and this review does not extend claims about them beyond that limited base.

### 4.2. Oral, Gastric, and Intestinal Digestion: What the Models Show, and Where They Disagree

Where digestion is simulated rather than measured in humans, the methodological standard is the INFOGEST static protocol, an international consensus method that fixes electrolyte composition, enzyme activities and pH for sequential oral, gastric (pepsin, low pH) and intestinal (pancreatin, bile salts) phases, developed because results from differing simulation conditions were often not comparable [43]. Outcomes nevertheless depend strongly on the scenario modeled. Applying three INFOGEST-consistent scenarios (infant, fed adult, fasted adult) to isolated lysozyme and ovalbumin and to food matrices containing them, one study found that the infant model left egg proteins largely intact whereas adult scenarios achieved much more extensive proteolysis, yet peptides longer than nine amino acids persisted through the intestinal phase in all three [44]. Standardization therefore does not guarantee comparable outcomes across scenarios, and even thorough adult digestion leaves peptides well above tripeptide length, which bears on how literally reports of intact, longer bioactive peptides reaching absorption should be read. Simulated age-related changes in gastric pH, enzyme concentration and digestion time likewise change how completely an egg preparation is broken down [42]. The egg-specific digestion studies cited here used static protocols. Static models do not reproduce gastric emptying, secretion kinetics, peristalsis or continuous product removal; dynamic systems such as the TNO gastrointestinal model (TIM), with dynamic gastric and intestinal compartments, and the Simulator of the Human Intestinal Microbial Ecosystem (SHIME), which couples digestion to a colonic microbiota, are designed to capture these features. Dynamic digestion of cooked whole egg with a peptidomic readout is therefore a gap (Section 13). Table 3 summarizes the effects of processing and digestion on egg-derived bioactives.

### 4.3. Enzymatic Release of Bioactive Peptides

Enzymatic hydrolysis—whether by digestive proteases during simulated or physiological digestion, or by exogenous enzymes such as alcalase or pepsin applied during food processing—is the mechanism that generates the bioactive peptides cataloged in Section 2.2 and Section 3 from their parent proteins in the first place [3,27]. The evidence reviewed in 4.1 indicates that release is not simply binary (peptide present or absent) but graded: the degree of prior heat treatment and the specific protease combination applied, pepsin alone versus pepsin followed by pancreatin, both shift the molecular weight distribution and the resulting antioxidant activity of the peptide pool obtained from egg white [40,41]. This bears directly on how egg-derived bioactive peptide research should be read: a peptide identified in a hydrolysate produced under one specific combination of enzyme, pH, and temperature is not automatically representative of what is released during ordinary human digestion of a cooked egg, and comparing bioactivity findings across studies that use different hydrolysis conditions risks comparing different peptide pools under a shared name.

### 4.4. Gastrointestinal Stability

Peptides do not survive the remaining digestive tract equally. A review of egg-derived peptides reports that specific antihypertensive sequences, including QIGLF, TNGIIR, RVPSL, RADHP, YAEER, LKP, IQW and YPI, resist further gastrointestinal digestion once formed, whereas others, such as FRADHPFL and YAEERYPIL, are further hydrolyzed [27]. This matters because in vitro bioactivity is physiologically relevant only to the extent that the peptide itself, rather than its amino acids, reaches the site of absorption or action. Gastrointestinal stability is also not a fixed property of a sequence: longer peptides persisted in every simulated scenario, but overall digestion completeness differed markedly between infant and adult models [44].

### 4.5. Bioaccessibility Versus Bioavailability

Bioaccessibility is the fraction of a compound released from the food matrix into the gastrointestinal lumen and therefore available for absorption; static models such as INFOGEST estimate it. Bioavailability is the smaller fraction that is absorbed, survives first-pass metabolism and reaches systemic circulation in an active form. A study of omega-3- and carotenoid-enriched eggs shows that compound classes behave differently under this distinction: linolenic fatty acid remained bioaccessible after digestion regardless of cooking method, lutein bioaccessibility generally decreased, and peptide content was essentially unchanged by the digestion step [45]. None of these findings establishes how much was subsequently absorbed (Section 4.6).

### 4.6. Absorption and Transport

**Figure 2:**
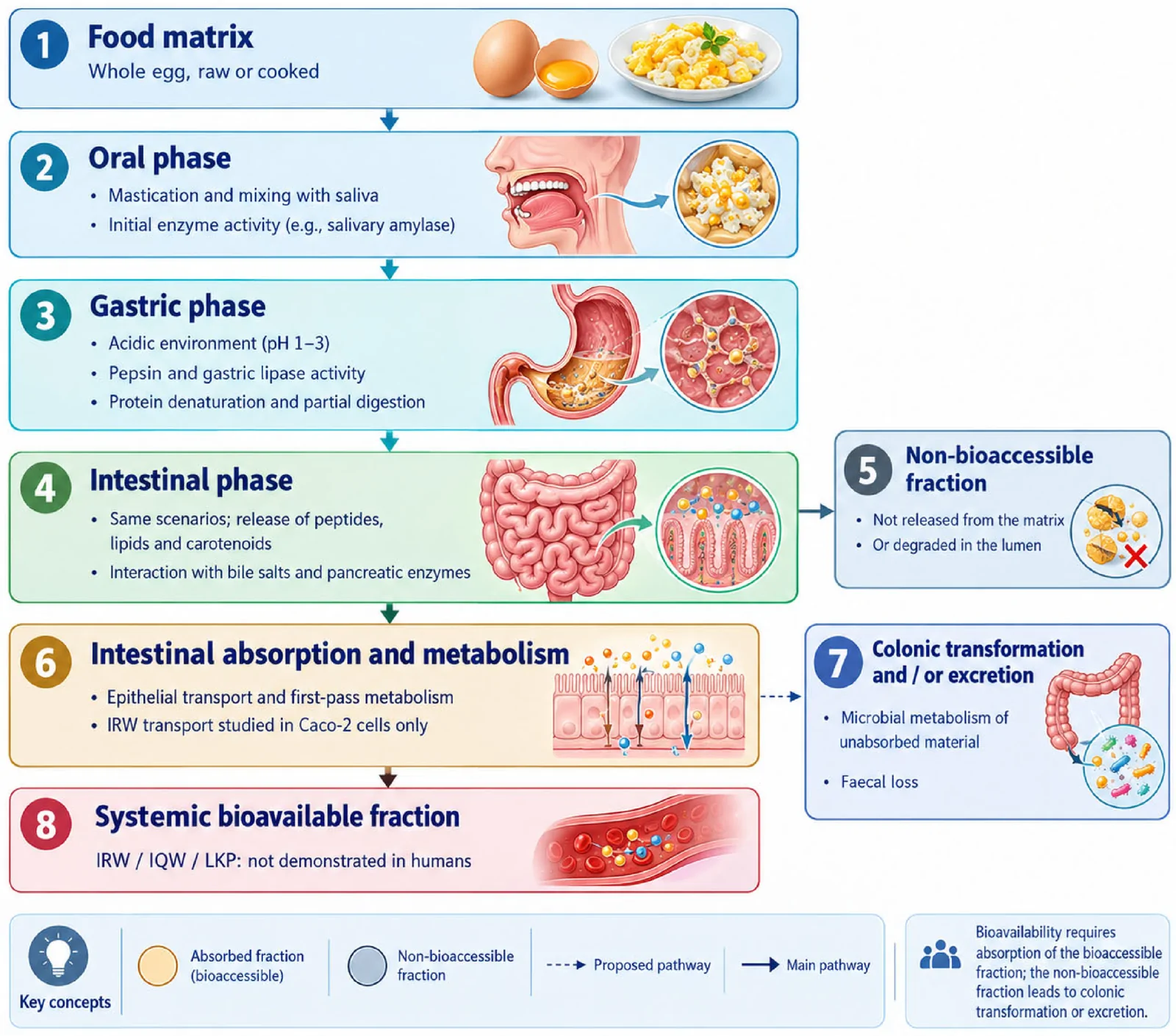
Digestion and bioaccessibility pathway. Figure generated with the assistance of ChatGPT (GPT-5.5; OpenAI, San Francisco, CA, USA); content reviewed, edited, and approved by the authors.

Compared with the digestion-and-release literature surveyed above, direct evidence on the intestinal absorption and systemic transport of egg-derived bioactive peptides specifically is sparse. The mechanistic routes proposed for peptide absorption in general—paracellular diffusion through tight junctions for very small peptides, transcellular passive diffusion, and carrier- or receptor-mediated transcytosis for larger or more structurally specific peptides—are described in the broader egg-peptide literature [27], but this review did not locate egg-specific human pharmacokinetic data confirming that any of the peptides cataloged in Section 2.2, including the mechanistically well-characterized ovotransferrin tripeptides IRW, IQW, and LKP, reach human systemic circulation intact after ordinary dietary consumption of eggs, at what concentration, or by which route. Transport of IRW across differentiated Caco-2 monolayers has been examined in vitro [46], but monolayer transport of a purified peptide does not address absorption in vivo from a digested food matrix. The IRW, IQW and LKP studies described in Section 2.2 used oral or intravenous administration to rodents and direct application to cultured cells [20,22,23], routes that bypass the absorption step, and none shows that absorption occurs at physiologically meaningful concentrations after ordinary egg consumption. In the terms defined in Section 2.2, these peptides are experimentally generated, not demonstrated. The pathway from the food matrix to systemic exposure, and the stages at which human evidence is missing, is summarized in Figure 2.

### 4.7. Post-Digestion Metabolites

Beyond intact peptide absorption, egg components can also act on the host indirectly through metabolites generated during or after digestion. The clearest documented example is compositional rather than mechanistic at this stage of the review: egg yolk phosphatidylcholine (Section 2.3) is a major dietary source of choline, whose further metabolism—including conversion to trimethylamine and its hepatic oxidation product trimethylamine-N-oxide (TMAO)—depends substantially on gut microbial activity rather than host digestion alone [11], linking this post-digestion metabolic step directly to the microbiome interactions taken up in Section 6 of this review. In keeping with the evidence-classification approach used in this review, this section notes the existence of that pathway without anticipating Section 6’s conclusions about its magnitude or health significance; the digestion-and-release evidence assembled here establishes only that the substrate—choline-rich phospholipid—is present, and that its downstream fate depends on processes occurring after the digestive steps covered in this section.

Taken together, the digestion literature supports three conclusions that carry forward. First, heat treatment is the only processing variable with direct human evidence, and that evidence rests on one small study [39]. Second, simulated-digestion outcomes depend as much on the physiological scenario modeled as on the food [42,44], so peptide pools reported under different conditions are not interchangeable [40,41]. Third, bioaccessibility has been measured for several compound classes, but the bioavailability of egg-derived peptides has not been measured in humans (Section 4.6); this is why the mechanistic findings in Section 5 are read as potential rather than demonstrated effects of egg consumption.

## 5. Egg-Derived Bioactives at the Intestinal Interface

This section addresses a narrower and more direct question than Section 2, Section 3 and Section 4: setting aside whether an egg-derived compound is generated, released, or absorbed in physiologically realistic amounts, what happens when it is placed in direct contact with intestinal epithelial cells? Here the evidence base, while still confined almost entirely to cell culture and occasionally rodent models, is more mechanistically specific than anywhere else in this review, because several studies go beyond reporting an endpoint—barrier integrity improved, inflammation reduced—to identify the tight-junction protein, signaling pathway, or cell-surface interaction actually responsible.

### 5.1. Barrier Integrity and Tight-Junction Proteins

The clearest direct evidence comes from the ovotransferrin tripeptide IRW, introduced in Section 2.2. In differentiated Caco-2 cell monolayers challenged with lipopolysaccharide (LPS), IRW pretreatment mitigated the LPS-induced fall in transepithelial electrical resistance (TEER, a standard measure of monolayer barrier integrity) and reduced paracellular permeability; mechanistically, IRW restored both the expression level and the normal cell-surface distribution of occludin, a core tight-junction protein whose mislocalization is a recognized marker of barrier dysfunction [24]. That is a meaningfully more specific claim than “IRW protects the gut barrier”: the study identifies the structural protein whose behavior IRW normalizes. A second, independent line of evidence reaches a barrier-protective endpoint from a different starting protein and a different challenge. Ovomucin hydrolysates, distinct from the ovotransferrin peptides above, restored TEER values in both human Caco-2 and porcine IPEC-J2 intestinal epithelial cell lines challenged with live enterotoxigenic *Escherichia coli* K88 (ETEC)—a pathogen-level challenge rather than a purified endotoxin challenge [29]. Beyond cell models, IRW and IQW administered to mice with dextran-sulfate-sodium (DSS)-induced colitis mitigated colonic inflammation and injury [47,48,49], extending the barrier-related evidence to an intact animal, although the peptides were again given as purified preparations rather than as part of a digested egg matrix. Together these findings, from two structurally unrelated egg proteins and two different challenge models, converge on the same qualitative endpoint—measurable, mechanism-linked protection of epithelial barrier integrity in cultured intestinal cells—though neither has yet been replicated in an intact intestinal tissue, organoid, or animal model with a physiologically dosed, digested food matrix rather than a purified peptide or hydrolysate applied directly to the apical cell surface.

### 5.2. Signaling Pathways: What Is Actually Shown, Not Merely Listed

For three points the studies above give direct support. First, IRW’s protection against LPS-induced barrier dysfunction is accompanied by inhibition of LPS-induced nuclear factor-κB (NF-κB) and mitogen-activated protein kinase (MAPK) activation in the same Caco-2 monolayers, together with a directly demonstrated LPS-neutralizing activity of the peptide, so part of its effect may occur upstream of the cell by inactivating the inflammatory trigger [24]. Second, ovomucin hydrolysates suppress ETEC-induced NF-κB and MAPK activation in both cell lines tested and also attenuate ETEC-induced activation of calcium/calmodulin-dependent protein kinase II (CaMKII), intracellular calcium elevation and oxidative stress [29]. Third, no study identified provides direct evidence, in an intestinal epithelial or gut-relevant model, implicating nuclear factor erythroid 2-related factor 2 (Nrf2), AMP-activated protein kinase (AMPK) or Toll-like receptor (TLR) signaling for an egg-derived compound. Nrf2 involvement is documented for food-derived antioxidant peptides as a class in other tissues, and TLR engagement is the expected route by which LPS is first sensed [24], but none was measured directly for an egg-derived compound in an intestinal cell model. For Nrf2, AMPK and TLR signaling, the appropriate reading is therefore plausible but unconfirmed, as opposed to directly demonstrated for NF-κB and MAPK. Table 4 summarizes the mechanisms linking egg-derived bioactives to intestinal, immune and metabolic responses.

### 5.3. Pathogen Adhesion: A Distinct Mechanism from Microbiome Composition Change

The ovomucin evidence illustrates a mechanism that should be separated from the microbiome-composition effects of Section 6. Ovomucin hydrolysates reduce ETEC K88 adhesion to intestinal epithelial cells, and this is substantially reduced when sialic acid residues are enzymatically removed, indicating that ovomucin’s sialylated glycans competitively interfere with the fimbrial adhesins ETEC uses to attach [29]. This is a host–cell-surface and pathogen-receptor interaction, not a demonstrated change in the resident community’s composition or metabolic output. Descriptions of egg-derived compounds as “antimicrobial” or “gut-protective” that do not distinguish this anti-adhesive mechanism from genuine microbiome modulation conflate two distinct claims; they are treated separately here.

### 5.4. Lysozyme and Mucosal Immune Interaction

As introduced in Section 2.1, hen egg white lysozyme’s interaction with gut-associated lymphoid tissue and intestinal lymphocyte populations following oral administration has been characterized, in a 2023 pharmacological review, as not fully dependent on the protein’s enzymatic (bacteriolytic) activity [19]. This is mechanistically distinct from the tight-junction and pathogen-adhesion evidence described above: rather than acting on the epithelial barrier or blocking a pathogen-adhesin interaction, this route implicates lysozyme, or peptidoglycan fragments generated when it digests luminal bacterial cell walls, as a direct immunomodulatory signal to gut-associated immune tissue. The review from which this characterization is drawn is itself a synthesis of pharmacological findings rather than a single new mechanistic experiment, and this review is careful not to overstate the granularity of the underlying evidence: the specific immune receptor or signaling pathway that lysozyme or its fragments engage at the gut mucosa was not resolved to the same level of mechanistic detail as the NF-κB/MAPK evidence in Section 5.2.

### 5.5. What This Section Does, and Does Not, Establish

Every mechanistic finding in this section came from purified peptides or hydrolysates applied directly to cultured cells (Caco-2, IPEC-J2) or, for lysozyme, from pharmacological and immunological observations after direct oral administration, not from digested whole-egg matrices delivering these compounds at the concentrations and molecular forms actually released during digestion (Section 4). The findings for IRW are therefore best read as what the peptide can do once it reaches an epithelial cell in sufficient concentration, not as evidence that this occurs during ordinary human digestion of eggs (Section 4.6). The same caveat applies, with somewhat less force given the higher molecular weight and demonstrated in vitro digestibility of ovomucin hydrolysates, to the anti-adhesive and barrier findings in Section 5.1 and Section 5.3. Connecting a digested, bioaccessible fraction of whole egg to a measured epithelial endpoint is a concrete research need (Section 13).

## 6. Egg-Derived Bioactives and the Gut Microbiome

This section addresses the link to the microbiome, where the gap between mechanistic plausibility and demonstrated human effect is widest. Two evidence streams must be kept separate throughout: a small human literature examining whole-egg consumption and microbiome-related outcomes directly, and a larger animal and cell-culture literature examining purified egg-derived peptides administered at defined doses. The two streams answer different questions, and this review resists the common move of citing findings from one to support claims properly belonging to the other.

### 6.1. Human Evidence: What Whole-Egg Consumption Studies Actually Show

A recent systematic review and meta-analysis [11] identified six human studies that examined egg consumption in relation to gut microbial composition or diversity, among twenty-two studies addressing egg intake and any gastrointestinal outcome. The six primary studies were consulted in their original reports and are summarized in Table 5: a four-week randomized controlled trial (RCT; crossover design) in 20 overweight postmenopausal women [13]; two cross-sectional studies using 16S rRNA gene sequencing, in 153 Italian adults [14] and 222 Korean adults [15]; and three studies using shotgun metagenomic sequencing, namely a cross-sectional study of 286 women in New Zealand [16], the Men’s Lifestyle Validation Study cohort of 307 US men [12], and the PREDICT 1 non-randomized intervention study of 1098 adults in the UK and USA [17]. The three studies using 16S rRNA sequencing found no significant association between egg intake and microbial composition or diversity, whereas each of the three using shotgun metagenomics reported significant associations [11]. Two features limit interpretation. First, in most of these studies egg intake was one of many dietary exposures examined rather than the primary exposure, so the studies were not designed or powered to detect egg-specific effects [11]. Second, sequencing method is confounded with design and population: the only randomized trial used 16S sequencing, and all shotgun studies were observational or non-randomized. Because shotgun metagenomics offers higher taxonomic resolution than 16S rRNA sequencing, the pattern is consistent with a real effect detectable only at higher resolution, but it is equally consistent with differences in design, population, exposure assessment or chance; the existing data cannot distinguish between these explanations.

Where associations were found, the specific taxa were not consistent across studies. *Bifidobacterium bifidum* was positively associated with habitual egg intake in one shotgun-metagenomic cohort [12] but negatively associated in another [16], and *Bifidobacterium adolescentis* was negatively associated in two studies [16,17]; this is not a coherent genus-level signal. More consistently, two of the three positive-association studies reported higher abundance of *Eubacterium* species, a genus containing major butyrate producers (*E. eligens* [17]; *E. rectale* [16]), and one of them additionally reported positive associations with *Akkermansia muciniphila* and *Faecalibacterium prausnitzii*, taxa widely discussed as beneficial, together with higher microbial evenness (Pielou’s index) among higher habitual egg consumers [16]. This is the closest this review comes to a human-supported claim about egg intake and specific beneficial taxa, but these are observational correlations in studies in which egg was one of many dietary variables; they do not show that egg consumption caused the taxonomic shift.

When the six studies are examined together across their key methodological characteristics, the pattern clarifies the limits of interpretation. The only RCT [13]—the study design that permits causal inference—used 16S rRNA sequencing, enrolled 20 participants, lasted 4 weeks and found no microbiome effect. All three studies reporting significant associations used shotgun metagenomics, but were observational or non-randomized, enrolled different populations across three countries, and quantified egg intake by habitual dietary instruments. Sequencing resolution, study design, population and exposure-assessment method are therefore confounded: the higher-resolution method co-occurs exclusively with the weaker causal design. Positive associations are consistent with a genuine microbiome signal 16S lacks the resolution to detect, but equally consistent with confounding by dietary pattern or lifestyle factors co-varying with egg intake.

The meta-analytic finding that whole-egg consumption raises plasma choline [11] reflects choline delivery to the bloodstream—a nutrient-delivery outcome, not evidence of an effect on gut microbiome composition or any mechanism attributable to egg-derived peptides. Similarly, microbiota-dependent TMAO formation from dietary phosphatidylcholine [35] describes a substrate-mediated metabolic pathway not mediated by egg-derived proteins or peptides; these findings should not be cited as evidence for gut-interface effects of egg bioactive peptides.

Beyond composition, fecal SCFA output was examined in relation to egg intake in only one human study, which found no significant association [14]. Circulating inflammatory markers, C-reactive protein (CRP) and cytokines including tumor necrosis factor-α (TNF-α) and interleukin-6 (IL-6), showed no consistent effect of egg consumption across randomized trials, and a pooled meta-analysis of three CRP studies found no significant effect [11]. Only one outcome showed a strong, consistent signal: plasma choline, which rose significantly after whole-egg intake in a meta-analysis of five short-term intervention trials (four weeks or less) [11]. The nutrient-delivery step of eating an egg, more choline entering the bloodstream, is therefore well established in the short term; every step downstream, toward microbiome composition, microbial metabolite production or measurable inflammatory consequence, is absent from the literature, based on a single study, or inconsistent.

### 6.2. The Choline–TMA–TMAO Axis: A Microbiota-Dependent Pathway with an Inconsistent Net Effect

Despite the inconsistency described above, the biochemical logic connecting egg-derived choline to a specific, microbiota-dependent metabolic pathway is comparatively well established, offering this review’s clearest example of a mechanism that is genuinely indirect—mediated by the microbiome rather than acting on it—while still being real. Dietary choline, including that supplied by egg yolk phosphatidylcholine (Section 2.3), is metabolized by specific gut bacteria to trimethylamine (TMA), which is absorbed and oxidized in the liver to trimethylamine-N-oxide (TMAO), a compound associated in other studies with cardiovascular and all-cause-mortality risk [11]. The microbiota dependence of choline-derived TMAO formation is supported by direct human evidence: in a phosphatidylcholine challenge (two hard-boiled eggs plus deuterium-labeled phosphatidylcholine) in healthy participants, suppression of the intestinal microbiota with oral broad-spectrum antibiotics markedly lowered the postprandial rise in plasma TMAO, which reappeared after antibiotics were withdrawn [35]; the experiment is also summarized in [11]. Because eggs were given together with a phosphatidylcholine supplement, this experiment establishes that the microbiota are required for TMAO formation from dietary phosphatidylcholine and choline, but it does not isolate egg-yolk phosphatidylcholine as the causal substrate, and it does not show that TMAO formation from eggs eaten alone is physiologically or clinically meaningful. Yet the net clinical relevance of this well-demonstrated mechanism remains genuinely unsettled at the level of ordinary egg consumption: the pooled meta-analysis found no significant increase in plasma TMAO after short-term whole-egg intake, several individual long-term cohort studies found significant positive associations between habitual egg intake and TMAO while others did not, and at least one cohort found that the answer depended on which dietary-assessment instrument (food-frequency questionnaire versus seven-day food diary) was used in the same study population [11]. A well-characterized mechanism and an inconsistent net human outcome are not in tension; they describe different things—the pathway’s existence, and its practical magnitude under realistic dietary conditions—and this review treats both facts as established without collapsing one into the other.

### 6.3. Direct Evidence from Purified Peptides: A Different Question, Answered More Clearly

Where purified egg-derived peptides rather than whole eggs are given to animals, evidence for an effect on gut microbial parameters is more consistent, though it addresses what an isolated, concentrated compound can do, not what ordinary food does. In a rat model of lipopolysaccharide-induced peritonitis, oral IRW significantly increased the Shannon diversity index and decreased the Simpson index of fecal microbiota relative to untreated LPS-challenged animals, a change the authors interpreted as consistent with support of intestinal barrier function [50]. In mice infected with *Citrobacter rodentium*, infection produced a phylum-level shift (lower Firmicutes and Actinobacteria, higher Bacteroidetes and Proteobacteria), but IRW and IQW did not significantly change overall diversity indices across the six groups, even though they influenced host serum metabolomic profiles [25]. In DSS-induced colitis in mice, oral IRW alleviated colonic injury, altered the abundance of Bacillota and other taxa and changed fecal SCFA levels [47], and dietary IRW and IQW increased antioxidant enzyme activities and a microbial diversity index while shifting several genera [48]. Together these studies show that a compound can influence host inflammatory and metabolic status without a detectable shift in microbial diversity, and that taxonomic change and host-relevant change are related but empirically separable outcomes: a study reporting one should not be read as having demonstrated the other. Table 6 summarizes the microbiome-related effects reported for purified egg-derived peptides and for the phosphatidylcholine–TMAO pathway.

### 6.4. Direct Versus Indirect Modulation: A Distinction This Review Maintains Throughout

Three mechanistically distinct routes by which an egg-derived compound could influence gut-microbiome-adjacent outcomes should be kept separate. First, direct modulation of microbial composition or metabolism (antimicrobial or prebiotic-like activity), for which the ovotransferrin and lysozyme mechanisms of Section 2.1 (iron restriction, peptidoglycan hydrolysis) provide plausible chemical bases, but for which no study identified demonstrates in situ modulation of a defined microbial community by a physiologically realistic, digested dose of egg-derived material. Second, receptor-level interference with pathogen adhesion, as shown for ovomucin hydrolysates against ETEC K88 (Section 5.3), which alters which organisms can colonize the epithelium without necessarily changing luminal community composition and is not “microbiome modulation” in the compositional sense. Third, indirect, substrate-mediated effects, in which digestion alters the substrate available to microbes; the choline–TMA–TMAO axis (Section 6.2) is the clearest example, alongside the general principle that increased substrate delivery to the distal gut can support microbial growth and diversity [11]. Statements that an egg-derived compound has “beneficial effects on the gut microbiome” should specify which route the evidence supports.

### 6.5. Methodological Limitations Across the Microbiome Literature

Several limitations beyond the 16S-versus-shotgun resolution difference bear on confidence in the microbiome findings. Cross-sectional and prospective cohort associations cannot show that egg consumption caused a taxonomic difference rather than being one correlate of a broader dietary or lifestyle pattern [11]. Taxonomic profiling describes which organisms are present, not what they are doing; paired metatranscriptomic or metabolomic data are largely absent from the egg-specific literature (Section 3.3 noted the corresponding gap for food composition). Even the most consistent finding, higher abundance of butyrate-associated genera with higher habitual egg intake [16,17], is therefore a compositional correlation, not a measured increase in butyrate production. The animal studies in Section 6.3 are interventional but rely on diversity indices (Shannon, Simpson) as proxies for a “healthier” community, and these indices do not indicate which functions are gained or lost.

## 7. Gut–Immune–Metabolic Interactions

This section does not introduce a large new evidence base. For the effects reported in Section 5 and Section 6, it asks whether the evidence supports a direct, microbiota-mediated, metabolite-mediated, host-mediated or multifactorial mechanism, and whether existing studies distinguish between these possibilities or only appear to.

### 7.1. A General Framework This Review’s Findings Could, in Principle, Connect to

A well-established literature outside the egg field describes how gut barrier dysfunction can drive systemic metabolic disease: when the mucus layer, tight junctions or epithelial restitution are compromised, lipopolysaccharide and other microbial products translocate into the circulation (metabolic endotoxemia), activating Toll-like receptor 4 and NF-κB signaling and contributing to insulin resistance, hepatic steatosis, adipose inflammation and vascular injury [51,52]. This is the host-side continuation of the barrier-integrity story in Section 5. A recent survey of natural bioactives that target this pathway discusses curcumin, resveratrol, quercetin, epigallocatechin gallate, berberine, anthocyanins, omega-3 fatty acids and dietary polysaccharides [51]; no egg-derived compound appears among its examples. The IRW and ovomucin findings in Section 5 involve analogous barrier-protective and NF-κB-modulating mechanisms, but egg-derived compounds have not been tested within the metabolic-endotoxemia framework to the extent of other dietary bioactives.

### 7.2. Classifying the Egg-Specific Effects Already Reviewed by Mechanism

Applying the direct/microbiota-mediated/metabolite-mediated/host-mediated/multifactorial distinction explicitly to findings already presented in this review is more informative than treating “gut–immune–metabolic interaction” as a single undifferentiated category.

The clearest case of a direct, host-mediated effect with no demonstrated gut or microbiome involvement is the ovotransferrin tripeptide IRW’s amelioration of angiotensin-II-stimulated insulin resistance in rat skeletal muscle cells [23] (see Section 2.2 of this review). That experiment applied IRW directly to cultured L6 myotubes; no intestinal epithelium, digestion step, or microbial community was present in the model, so however this finding is eventually contextualized, it should not be described—as reviews of egg bioactives sometimes do, by proximity of subject matter—as a “gut-mediated” metabolic effect. It is evidence of a direct cellular mechanism, and its relevance to this review’s central pathway depends entirely on the unresolved absorption question raised in Section 4.6: whether IRW reaches muscle tissue intact after digestion of whole egg, not on anything happening in the gut.

The barrier-protective and NF-κB-inhibiting activity of IRW and of ovomucin hydrolysates in Caco-2 and IPEC-J2 cells (Section 5.1 and Section 5.2) is also direct and host-mediated in the specific sense that the compounds act on epithelial cells themselves, but it is mechanistically continuous with the metabolic-endotoxemia framework in Section 7.1: both studies used exogenous LPS or live pathogen challenge to simulate what a compromised barrier would allow through, rather than demonstrating that the compound prevents endogenous barrier compromise arising from an unrelated insult—a high-fat diet, for instance—followed by reduced endotoxin translocation and downstream metabolic improvement. This is a meaningful gap between what has been shown (protection against an experimentally imposed challenge) and what the broader metabolic-endotoxemia literature would consider the more clinically relevant demonstration (prevention of barrier compromise arising under realistic dietary or pathological conditions).

The choline–TMA–TMAO pathway (Section 6.2) is this review’s clearest example of a genuinely metabolite-mediated, microbiota-dependent effect, established through the antibiotic-ablation evidence discussed there [11]. Its distinguishing feature is that the egg-derived compound (choline) never itself acts on the microbiome or the host directly; it is a substrate for a microbial transformation whose product then acts on the host.

The IRW peritonitis findings (Section 6.3) are best classified as multifactorial and mechanistically underdetermined. The study observed both reduced systemic inflammatory markers and altered fecal diversity indices in the same LPS-challenged rats [50] but did not establish the direction of causality: IRW could act directly on host inflammatory signaling (consistent with its NF-κB-inhibiting activity in epithelial cells, Section 5.2), with the microbiota shift a downstream consequence; it could act partly through the microbiota, which then feeds back on host inflammation; or both could be independent, parallel effects. The *Citrobacter rodentium* study (Section 6.3), in which IRW and IQW affected host serum metabolomic profiles without a significant change in diversity indices [25], is mild evidence against microbiota mediation being necessary for the host-level effects of this peptide family, although one non-significant result in one infection model does not exclude microbiota involvement.

### 7.3. Oxidative Stress as a Recurring but Mechanistically Heterogeneous Theme

Oxidative stress modulation appears repeatedly in this review, but not through a single shared mechanism. The antioxidant activity of ovotransferrin and phosvitin (Section 2.1 and Section 2.3) is chemical, resting on iron chelation that limits iron-catalyzed oxidation. Ovomucin hydrolysates attenuate ETEC-induced oxidative stress in intestinal epithelial cells downstream of CaMKII activation and calcium elevation [29] (see Section 5.2 of this review), a signaling-linked rather than radical-scavenging mechanism. Lysozyme’s mucosal immune interactions [19] (see Section 5.4 of this review) have not been tied to oxidative stress modulation. Grouping these under one “antioxidant” heading obscures at least three distinct routes, each supported by different evidence. No study identified tested whether an egg-derived bioactive, delivered as part of digested whole egg, reduces intestinal permeability or circulating endotoxin markers under barrier compromise; the gut–immune–metabolic connections therefore remain a coherent hypothesis built from adjacent but unconnected evidence.

## 8. From Bioactivity to Prospective Precision Nutrition

Precision nutrition is considered here as a prospective application. The question is whether two people eating the same egg would be exposed to the same bioactive compounds, and whether tools for measuring individual variability can currently support egg-specific recommendations. Section 8.1 lists potential modifiers of response; Section 8.2 explains why the framework remains one of research rather than practice.

### 8.1. Potential Modifiers of Response: General Evidence, Not Egg-Specific

Four factors could plausibly modify exposure to, or response to, egg-derived compounds. For none of them do the studies reviewed here show that the factor modifies the response to egg-derived bioactives in humans; they are potential modifiers relevant to an egg-specific hypothesis, and the supporting evidence comes from non-egg or simulated contexts. (i) Age: a simulated elderly gastrointestinal model reduced proteolysis of omelet-prepared egg by up to 37% relative to a standard adult model, with the deficit varying by cooking method [42]; this is an in vitro finding, not a human measurement. (ii) Flavin-containing monooxygenase 3 (FMO3) genotype and renal status: common FMO3 variants, which affect hepatic oxidation of microbially generated TMA to TMAO, are associated with circulating TMAO concentrations in a dose-dependent manner with the number of variant alleles in patients with chronic kidney disease (CKD) [53]; this association was not studied in relation to egg intake. (iii) Sex: in a multi-ethnic population cohort (n = 5166), sex was only modestly associated with gut-microbiome composition (area under the curve [AUC] 0.61–0.63, where 0.5 indicates no discrimination), and much of the raw association was attributable to correlated factors such as age, cardiovascular risk and diet [54]; this too is not egg-specific. (iv) Habitual diet and health status: dietary pattern and baseline microbiome were contextual factors in the human egg–microbiome studies [11], but none tested them as modifiers of the response to eggs. Dose, frequency, food matrix and processing are further sources of variation: human egg interventions covered a narrow range (one to four eggs per day for up to twelve weeks) [11], and cooking method and compound class changed digestion outcomes in simulated systems [42,45].

### 8.2. A Prospective Research Framework, Not Clinical Practice

Precision nutrition is treated here as a prospective research framework for egg-derived bioactives, not an established strategy. The FMO3–choline–TMAO axis supports one testable hypothesis: that carriers of variant FMO3 alleles, particularly those with reduced renal clearance, may differ in TMAO exposure after equivalent egg intake. This is a legitimate research question but not a basis for clinical recommendation, because the prior question, whether egg-derived TMAO exposure at ordinary dietary levels has a health consequence in generally healthy people, remains unresolved [11]. The computational tools proposed for personalization are also not yet validated for this purpose: peptide-prediction pipelines needed cellular and animal confirmation [26,38], the largest microbiome–sex analysis showed modest discrimination [54], and general assessments conclude that microbiome data cannot yet be translated into precise dietary advice for healthy individuals without corroborating biomarkers [7,8]. Precision-nutrition frameworks are therefore downstream of the basic evidentiary questions identified in this review, not a way of resolving them. Translating current evidence into egg-specific precision-nutrition guidance or functional-food applications would require, at minimum: (i) human pharmacokinetic data confirming that the relevant compound reaches the systemic circulation from ordinary whole-egg consumption; (ii) dose–response data in humans; and (iii) independent safety data distinct from efficacy experiments. None of these prerequisites is currently met for any egg-derived bioactive peptide reviewed here.

## 9. Delivery Systems and Functional Food Applications

Delivery-system engineering could in principle address the gastrointestinal-stability problem identified in Section 4 and Section 5, but its application to egg-derived bioactives is limited, and it is covered here only briefly. Most of the egg-related delivery literature runs in the opposite direction, using egg white proteins (notably ovalbumin) and yolk lipids as encapsulants for non-egg compounds [55,56], and is outside the scope of this review. One example is directly relevant: yolk immunoglobulin Y (IgY) encapsulated in liposomes made from yolk-derived phosphatidylcholine was designed to resist gastric inactivation, with an encapsulation efficiency of 76.85% [57]. Egg white protein nanoparticles have likewise improved the heat and trypsin stability of the non-egg peptide nisin [58]. A 2026 study of self-immolative peptide–prodrug conjugates for inflammation-targeted oral delivery included IRW alongside two other anti-inflammatory peptides and reported gastrointestinal stability and benefit in colitis mice [59]; that platform relies on synthetic polymer chemistry rather than egg-derived carriers, and no human data are available. We identified no delivery-system study for IQW, LKP or the ovomucin hydrolysates, and no cost, scalability or regulatory analysis for any egg-specific delivery system. Two constraints apply: the leading egg encapsulants (ovalbumin and ovotransferrin) are themselves major allergens [33,55] (Section 10), and the laboratory fabrication methods used (acid–heat treatment at 90 °C; supercritical CO_2_ at 20 MPa) have not been evaluated at food-manufacturing scale [57,58]. Delivery engineering is therefore a laboratory capability, not a validated route to functional-food application for egg-derived bioactives.

## 10. Safety, Allergenicity, and Regulatory Considerations

Safety is a necessary counterpart to bioactivity in egg: several of the egg proteins with the strongest claim to gut-relevant bioactivity in this review—ovalbumin, ovotransferrin, and, to a lesser degree, ovomucin and lysozyme—are simultaneously among the proteins most responsible for egg’s status as a major food allergen. Bioactivity and allergenicity are not competing considerations to be weighed against each other in the abstract; in egg, they frequently attach to the same molecules.

### 10.1. Egg Allergy: Scope and the Allergens Involved

Egg is among the most common food allergens in children worldwide, with prevalence reported as high as 10%; most affected children lose clinical reactivity by later childhood, but a minority carry the allergy into adulthood [33]. Five proteins are recognized as major hen’s egg allergens: ovomucoid (Gal d 1), ovalbumin (Gal d 2), ovotransferrin (Gal d 3) and lysozyme (Gal d 4) from egg white, and α-livetin (Gal d 5) from yolk [33,60]. Four of the five proteins most discussed in Section 2.1 are therefore designated clinical allergens. Ovomucin, the source of the anti-adhesive, barrier-protective hydrolysates of Section 5.1 and Section 5.3, is not among the four principal egg white allergens, and its allergenic potential was not examined in the sources retrieved for this review. Any functional-food application built on these proteins or their hydrolysates inherits this allergenic burden as a starting condition.

### 10.2. Effects of Processing on Allergenicity: Uneven, and Allergen-Specific

Processing does not reduce all egg allergens equally. Ovomucoid is exceptionally thermostable and resistant to digestion, which makes it the dominant allergen, the one to which most egg-allergic patients are sensitized, despite ovalbumin being present in greater quantity [33]. Because ovalbumin’s epitopes are more heat-labile, some individuals allergic mainly to ovalbumin tolerate extensively heated (for example, baked) egg, whereas ovomucoid-sensitized individuals more often do not [33]. Processing effects on the yolk allergen α-livetin have been examined less extensively and appear processing-dependent [60]. Any claim that cooking “reduces allergenicity” is therefore allergen-specific: the heat treatment shown in Section 4.1 to improve egg protein digestibility does not equivalently neutralize ovomucoid.

### 10.3. Bioactive Peptide Safety: An Evidence Gap This Review States Plainly

Despite the mechanistic depth documented for IRW, IQW and LKP (Section 2.2, Section 5, Section 6 and Section 7), no dedicated toxicological or safety assessment of these peptides was identified: no defined no-observed-adverse-effect level, no systematic dose-escalation study, and no human safety trial distinct from the efficacy studies. The rodent and cell studies were designed to test efficacy and mechanism, and the absence of reported adverse effects in an efficacy study is not a systematic safety assessment. This gap is continuous with the dose-and-frequency gap in Section 8.1: without safe dosing ranges for these peptides and without human data at the doses used in rodents, translating IRW’s rodent activity into a human dosing recommendation would go beyond the evidence.

### 10.4. A Documented Interaction Distinct from Allergenicity: Avidin and Biotin

Separately from allergenicity, one egg white protein carries a well-established nutrient-interaction risk. Avidin, a minor egg white glycoprotein, binds biotin with one of the strongest known non-covalent interactions, and the complex resists digestive proteolysis, preventing biotin absorption when sufficient raw avidin is consumed. A classic pediatric case report documented biotin deficiency (alopecia, dermatitis and impaired leukocyte carboxylase activity) in a child whose diet included substantial raw egg, with full reversal after biotin supplementation and removal of raw egg [34]. As with allergenicity, the risk is heat-dependent: cooking destroys avidin’s biotin-binding capacity, so the interaction is associated almost exclusively with large, prolonged intakes of raw or minimally heated egg white. It is included because a safety section limited to allergenicity would omit a mechanistically well-characterized food-safety consideration specific to egg.

### 10.5. Translational Note on Regulation and Health-Claim Evidence

Regulatory pathways for peptide ingredients, hydrolysates and health claims differ among jurisdictions (for example, the European Union, the United States, Canada and Asian countries). This review does not analyze or compare them, and this section is a general translational discussion rather than a regulatory review; no regulatory documents were assessed, and no claim is made about the regulatory status of any specific egg-derived peptide, hydrolysate or delivery system. What the evidence synthesized here does indicate is the kind of data any jurisdiction would be expected to require before a health claim for a specific egg-derived bioactive could be considered: a demonstrated effect at ordinary dietary exposure, in humans, on a defined health outcome (Section 8.2 and Section 11), together with safety data independent of the efficacy studies (Section 10.3). For the compounds followed most closely here, the ovotransferrin peptides and the choline–TMAO axis, such data are currently lacking.

## 11. Human Evidence and Translational Potential

### 11.1. Evidence Matrix for the Central Findings

This section summarizes the evidence mapped in this review using seven translational evidence categories: Category 1, in silico prediction; 2, chemical or biochemical assay; 3, in vitro cellular study; 4, simulated gastrointestinal digestion; 5, animal study; 6, human observational study; 7, human intervention or clinical studies. These categories are a heuristic classification of the experimental system used, not a validated hierarchy and not an ordinal scale of evidence strength. They answer different questions (a simulated-digestion study addresses release and stability, which a cell study cannot); a well-designed cell experiment can be more informative about mechanism than a weak human study, and a human pharmacokinetic study and a randomized efficacy trial answer different questions although both fall in Category 7. Study quality is therefore appraised separately (Section 1.1), and findings are summarized in a multidimensional evidence matrix across eight dimensions: molecular identification, digestive release and stability, intestinal transport and bioavailability, mechanism, animal efficacy, human exposure and pharmacokinetics, human efficacy, and safety.

The evidence matrix in Table 7 maps the eight dimensions of evidence for each major egg-derived bioactive compound. Three epistemic states must not be conflated: (a) an uninvestigated question—no study designed to address this dimension was found in the search; (b) a gap in retrieved evidence—studies may exist in databases not searched; (c) a confirmed negative finding—a well-designed study returned a null or negative result. For all human-evidence dimensions in Table 7, all N entries represent states (a) and/or (b); no example of state (c) was found for any peptide-level dimension. The evidence categories (Categories 1–7) describe the experimental system used, not a quality hierarchy.

### 11.2. Where Human Evidence Genuinely Exists

Human evidence in this review clusters narrowly. It is strong and consistent for one finding: whole-egg consumption raises plasma choline in short-term trials [11]. It is real but more limited for two others: the microbiota dependence of TMA/TMAO formation after eggs plus phosphatidylcholine, shown by antibiotic suppression in healthy participants [35], and the higher true ileal digestibility of cooked than raw egg protein in five ileostomy patients [39]. Beyond these, human evidence is observational and inconsistent (egg intake and microbiome composition, TMAO, inflammatory markers; Section 6.1) or associative and not specific to egg (FMO3 genotype, sex; Section 8.1). Clinical evidence on egg allergenicity is abundant (Section 10.1 and Section 10.2) but addresses sensitization and reactivity, a different question from gut bioactivity, and the two bodies of evidence should not be conflated.

### 11.3. Where Human Evidence Is Entirely Absent

The pattern visible in Table 7 is that the compounds with the deepest mechanistic characterization, the ovotransferrin tripeptides IRW, IQW and LKP and the ovomucin hydrolysates, have no human evidence at any point along the pathway from digestion to effect: no human pharmacokinetic study confirms that these peptides are generated during digestion of egg (Section 4.6), and every mechanistic claim about them (ACE inhibition, ACE2-axis activation, insulin sensitization, tight-junction restoration, NF-κB/MAPK inhibition, anti-adhesion, effects on microbiota diversity) rests on Category 2, 3 or 5 evidence. This contrasts with the choline–TMA–TMAO pathway, which is mechanistically characterized and has been tested in humans: its mechanism is established and its net health relevance is uncertain (Section 6.2), whereas for the ovotransferrin peptides both relevance to ordinary egg consumption and net health relevance are uncertain, because the prior step, whether the peptides reach an intestinal cell or the circulation in active form, has not been tested. In vitro and animal findings for peptides such as IRW should therefore not be stated in the same declarative register as the human choline–TMAO data.

## 12. Contradictions, Limitations, and Knowledge Gaps

Specific limitations were flagged as they arose. This section groups them by origin and does not repeat points already made in Section 4.6, Section 5.5 and Section 11.

### 12.1. Contradictory Findings, and Their Methodological Origin

Four apparent contradictions recur. (i) Egg intake’s association with *Bifidobacterium* differed by species and study: *B. bifidum* was positively associated in one cohort [12] and negatively in another [16], and *B. adolescentis* negatively in two [16,17], so there is no coherent genus-level signal. (ii) All studies using 16S rRNA sequencing found no significant microbiome association with egg intake, whereas all using shotgun metagenomics did (Section 6.1); sequencing resolution may explain part of this, but design, population and exposure assessment are confounded with sequencing method. (iii) The association of egg intake with TMAO depended, in one cohort, on whether intake was measured by food-frequency questionnaire or seven-day food diary [11] (see Section 6.2 of this review). (iv) IRW changed fecal diversity indices in a rat peritonitis model [50] and in DSS colitis [48], but not in a mouse *Citrobacter rodentium* infection model [25], a difference plausibly related to host species, challenge and infection biology. In each case the contradiction is reduced once the measurement method or model system is taken into account, which is why methods are reported alongside findings.

### 12.2. Digestion Protocols, Egg Source, and Standardization

Even a standardized protocol (INFOGEST) yields materially different outcomes depending on the physiological scenario simulated [44], and heat pretreatment and enzyme combination jointly determine the peptide pool [40,41]. Studies reporting “the peptides released from egg white during digestion” are therefore often not describing the same pool, and standardizing the protocol [43] does not standardize which scenario a study models.

Egg composition also varies with breed and diet: a controlled comparison of an indigenous and a commercial hybrid breed found different fat (9.53% versus 7.58%) and protein (12.31% versus 11.66%) contents, with diet further affecting the fatty acid profile [61]. In the studies cited here, hen breed, husbandry system and diet were generally not reported, so compositional variation cannot be excluded as a source of inconsistency.

Finally, peptides confirmed by mass spectrometry differ in evidential standing from prediction-only candidates (Section 3), and no dosing standard links the one-to-four-egg human interventions [11] to the peptide doses used in rodent and cell experiments; no dose–response relationship has been established for any characterized peptide.

### 12.3. Microbiome Heterogeneity, Trial Duration, and Translation to Food Matrices

Sequencing method, study design and the near absence of paired metatranscriptomic or metabolomic data limit functional interpretation (Section 6.5); with only six human microbiome studies, heterogeneity across two or three studies can decide whether the field appears consistent or inconsistent. Human evidence is also split between short intervention trials (up to twelve weeks) and observational studies of habitual, long-term intake [11]. A mismatch of time scales could explain part of the inconsistency (positive associations with beneficial taxa came from habitual intake, null TMAO and inflammatory findings from short trials), but no study was designed to test this, so it remains a hypothesis, and long-term efficacy and safety are extrapolations beyond the evidence. Finally, the compounds with the richest mechanistic evidence have been studied almost exclusively as purified preparations in cells or animals, not as components of digested whole-egg matrices at realistic doses (Section 5.5), which is the limitation beneath most of the others.

## 13. Future Research Priorities

Each priority below is linked to a gap identified in Section 2, Section 3, Section 4, Section 5, Section 6, Section 7, Section 8, Section 9, Section 10, Section 11 and Section 12 between laboratory evidence and human relevance. These priorities assume that prerequisite steps—particularly human pharmacokinetic confirmation that named peptides reach the circulation from ordinary egg consumption—have not yet been accomplished. Designs are illustrative examples, not preferred protocols; doses, durations and group sizes require justification by pilot data and power calculations. Priorities are grouped by the time frame over which they could realistically be executed with current methods, not by importance.

### 13.1. Short-Term Priorities (1–3 Years)

**Targeted peptidomic screening of whole-egg digests for named bioactive sequences.** No study retrieved here has confirmed that IRW, IQW or LKP are generated when intact, cooked whole egg undergoes digestion (Section 4.6). One illustrative design would apply the INFOGEST protocol [43] across infant, adult and elderly-adjusted scenarios [42,44] to cooked whole egg, with targeted liquid chromatography–tandem mass spectrometry (LC-MS/MS) peptidomics for these sequences at each digestive phase; dynamic systems such as TIM or SHIME could extend this.

**Testing purified-peptide findings against real digests.** The barrier-protective activity of IRW and ovomucin hydrolysates has been shown only with purified preparations (Section 5.5). The LPS and ETEC challenge assays [24,29] could be repeated with the intestinal-phase output of whole-egg digestion, at concentrations matched to those found by the peptidomic screen, to test whether the effect persists with a real digestate.

**Reporting of hen source variables.** Breed, diet and husbandry system were generally not reported in the studies cited (Section 12.2) despite documented effects on egg composition [61]; reporting them alongside compositional or bioactivity findings would allow future analyses to test whether they explain heterogeneity.

### 13.2. Medium-Term Priorities (3–5 Years)

**A human pharmacokinetic study of the ovotransferrin tripeptides.** This is the study that would most advance the field: healthy volunteers receive a defined portion of cooked whole egg (or an IRW-enriched preparation, once screening establishes feasible concentrations), and plasma and urinary IRW, IQW and LKP are measured by targeted LC-MS/MS over a postprandial time course. A positive result would provide the first human exposure data for this peptide family; a negative or below-detection result would be equally informative, redirecting the mechanistic literature toward effects at non-physiological, directly administered doses.

**Longer human trials with shotgun metagenomic sampling.** To test the possible time-scale mismatch (Section 12.3), an intervention lasting several months, with shotgun metagenomic sampling at baseline and several follow-up points rather than 16S rRNA sequencing [11], could show whether null short-term TMAO and inflammatory-marker findings reflect no effect or a slowly accumulating one.

**Paired functional readouts.** Egg-intervention microbiome studies should pair taxonomic sequencing with metatranscriptomic or targeted metabolomic measures (for example, fecal SCFA and bile acids, measured in only one existing study [14]) so that compositional shifts can be evaluated for functional relevance (Section 6.5 and Section 12.3).

**Dose–response characterization.** As no dose–response relationship has been established for any characterized peptide or for egg-derived outcomes (Section 12.2), an illustrative design would vary whole-egg intake across several levels (for example, none, one, two and four eggs per day, the range already used in human trials [11]) with a standardized hen source, measuring plasma choline, TMAO and, when available, peptide markers in parallel.

**Dedicated toxicological assessment.** No safety study independent of efficacy testing was retrieved for IRW, IQW, LKP or the ovomucin hydrolysates (Section 10.3); a standard toxicological evaluation, including dose escalation and determination of the no-observed-adverse-effect level, would be a prerequisite for any functional-food application.

### 13.3. Long-Term Priorities (5–10 Years)

**A test of the precision-nutrition hypothesis for egg bioactives.** Section 8.2 concluded that this remains a prospective framework. A long-duration, standardized whole-egg intervention in participants stratified by FMO3 genotype [53] and renal function (and, if justified, baseline microbiome composition) could test whether strata differ in TMAO or microbiome responses to identical egg exposure. FMO3 genotype is appropriate here as a stratification variable or effect modifier.

**An egg-specific bioaccessibility and bioavailability reporting standard.** A consensus reporting approach building on INFOGEST [43] would permit cross-study comparison of bioaccessibility and bioavailability data for egg bioactives (Section 12.2).

**Better exposure assessment in observational egg–microbiome studies.** Habitual-intake associations remain confounded by broader dietary pattern (Section 6.5). Prospective cohorts with repeated dietary assessment and repeated shotgun metagenomic sampling could reduce confounding. FMO3 variants are not suitable Mendelian-randomization instruments for egg intake, because FMO3 influences TMAO derived from many dietary substrates and could affect TMAO independently of egg consumption; they are better used as effect modifiers or stratification variables, or as instruments in studies of TMAO metabolism itself. Table 8 summarizes the gaps and illustrative research approaches.

## 14. Discussion

**Figure 3:**
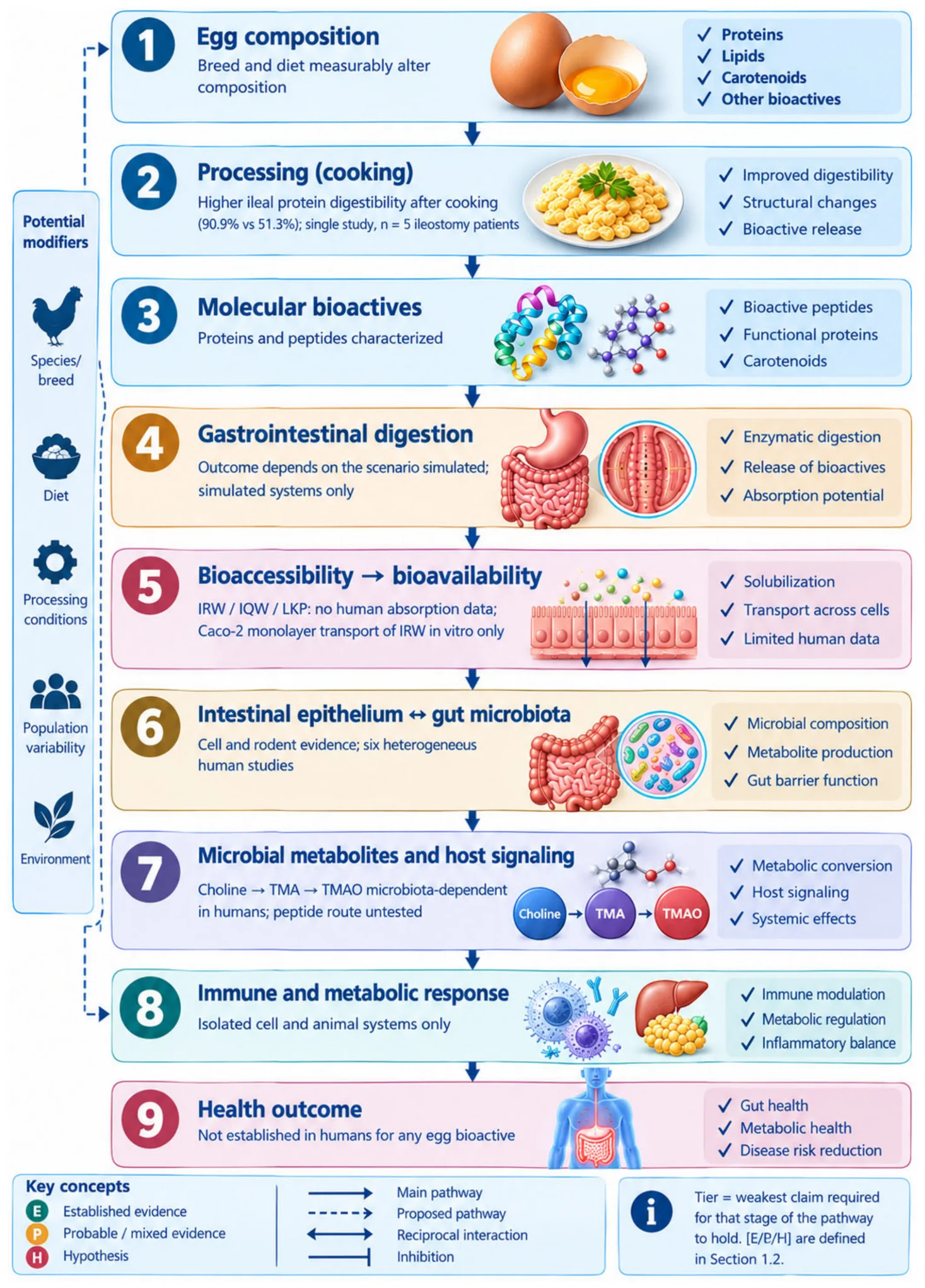
Egg-derived bioactives at the gut interface. Numbers 1–9 denote the successive stages of the pathway. Evidence tiers [E], [P] and [H] are defined in Section 1.2, and the potential modifiers shown at the left are discussed in Section 8.1. Main sources for each stage: 1 [61]; 2 [39]; 3 [1,3,4,20]; 4 [42,44]; 5 [46]; 6 [12,13,14,15,16,17,25,47,48,50]; 7 [35]; 8 [19,20,22,23,24,29]; 9 [11]. Figure generated with the assistance of ChatGPT (GPT-5.5; OpenAI, San Francisco, CA, USA); content reviewed, edited, and approved by the authors.

The pathway traced in this review is summarized in Figure 3 using the established [E], probable [P] and hypothesis [H] tiers defined in Section 1.2. The tiers describe the status of each link for egg-derived compounds; general physiological evidence extrapolated from non-egg contexts is not classed as [E]. The panel at the left of Figure 3 shows the individual factors that could modify the pathway; these are labeled as egg-specific evidence or general evidence extrapolated to egg, and none has been shown to modify the response to egg bioactives (Section 8).

Read from composition to outcome, Figure 3 shows that support for the pathway is not continuous. Egg-specific [E] evidence is confined to composition and characterization (Section 2 and Section 3), the effect of cooking on protein digestibility (one small human study [39]), short-term plasma choline responses to whole-egg intake [11], and the microbiota dependence of choline-derived TMAO formation [35]. Cell and rodent evidence supports [P] mechanisms for IRW and ovomucin hydrolysates at the epithelium (Section 5), but the links that would connect them to human egg consumption (formation and survival of the peptides during digestion, absorption, and effects on host outcomes) are [H].

Activities should not be generalized across compounds. IRW and IQW share anti-inflammatory and antioxidant activity in endothelial cells, whereas only IRW improved insulin resistance in muscle cells and LKP did not [21,23]; ovomucin hydrolysates act through sialylated-glycan-dependent anti-adhesion [29]; the immunomodulatory effect of lysozyme is not fully dependent on its enzymatic activity [19]; and the choline pathway acts through microbial conversion of a substrate [11,35]. These are mechanistically distinct (direct epithelial, anti-adhesive, immune and substrate-mediated; Section 5, Section 6 and Section 7) and should be reported as such.

The human-evidence findings agree with a recent systematic review of whole-egg clinical studies [11], which found conflicting results for most gastrointestinal outcomes except plasma choline, and extend it by showing that the molecular literature on egg-derived peptides has not been connected to human exposure. Earlier reviews have cataloged the barrier- and microbiota-related benefits of egg white proteins and peptides [10]; the present review adds a separation of evidence by experimental system and a mapping onto human data.

For product development, the evidence supports cooking as a way to improve egg protein digestibility (based on one small human study), supports eggs as a choline source, and supports further development of IRW, IQW and ovomucin hydrolysates only as a hypothesis pending human exposure data. For precision nutrition, FMO3 genotype, renal function, age and sex are plausible modifiers that remain untested for egg bioactives (Section 8). The limits of this review are those of its method: a narrative synthesis with a documented but non-systematic search of two databases, PubMed/MEDLINE and Europe PMC (Section 1.1; Scopus and Web of Science were not searched), no formal risk-of-bias scoring of primary studies, and reliance on one systematic review [11] to frame the human evidence. Statements that no study was identified refer to this search and may be revised by newer work.

## 15. Conclusions

This review traced one pathway (egg composition, molecular characterization, digestion, bioaccessibility, intestinal and microbial interaction, host signaling and prospective precision nutrition) and classified the evidence for each link by experimental system.

Three findings are supported in humans: cooked egg protein had higher true ileal digestibility than raw egg protein in a small ileostomy study (n = 5) [39]; whole-egg intake raises plasma choline in short-term trials [11]; and gut microbiota are required for TMAO formation after eggs plus phosphatidylcholine [35]. In cells and animals, IRW, ovomucin hydrolysates and, to a lesser extent, IQW and lysozyme show compound-specific effects on barrier function, inflammatory signaling, anti-adhesion and immune modulation (Section 5, Section 6 and Section 7).

No study identified shows that IRW, IQW or LKP are formed, survive digestion or reach the circulation after ordinary consumption of cooked whole egg, and the human microbiome literature on egg intake comprises six heterogeneous studies in which egg was mostly one of many exposures [12,13,14,15,16,17]. The net health relevance of egg-derived TMAO at ordinary intakes is unsettled [11]. Precision nutrition, delivery systems and regulatory pathways for egg bioactives remain prospective.

The priorities that follow are human pharmacokinetic confirmation of the ovotransferrin tripeptides from cooked whole egg, longer trials with shotgun metagenomic and functional readouts, and independent safety evaluation (Section 13). Until such data exist, mechanistic findings for egg-derived peptides should be read as potential, not demonstrated, effects of egg consumption.

## Figures and Tables

**Table 1:** Summary of bioactive peptides, their reported bioactivities and evidence categories from egg proteins.

Peptide/Sequence	Parent Protein	Reported Activity	Experimental Model	Evidence Category	Ref.
IRW (Ile-Arg-Trp)	Ovotransferrin	ACE inhibition; antihypertensive via ACE2/Ang-(1-7)/Mas axis; anti-inflammatory; barrier-protective; insulin-sensitizing	SHR rat (oral); Caco-2 cells; L6 rat myotubes	Category 3 (cell)/Category 5 (SHR rat)	[20,22,23,24]
IQW (Ile-Gln-Trp)	Ovotransferrin	ACE inhibition; anti-inflammatory and antioxidant in endothelial cells; no improvement of insulin resistance in L6 myotubes; tested with IRW in *C. rodentium* mouse model	Human umbilical vein endothelial cells; L6 rat myotubes; *C. rodentium*-infected mice	Category 3 (cell)/Category 5 (mouse)	[21,23,25]
LKP (Leu-Lys-Pro)	Ovotransferrin	ACE inhibition; did not replicate the IRW effect on insulin resistance in L6 myotubes	Enzyme assay; L6 rat myotubes (activity not replicated)	Category 2; Category 3 (not replicated)	[23]
AAQEKIRYW (Ala-Ala-Gln-Glu-Lys-Ile-Arg-Tyr-Trp)	Egg yolk protein (alcalase and pepsin digests)	Dipeptidyl peptidase-4 (DPP4) inhibition, IC_50_ 36.65 µM	Machine-learning/docking prediction → cell assay → in vitro digestion → mice (oral glucose tolerance test in healthy mice; plasma DPP4 activity in type 2 diabetic mice)	Categories 1, 3, 4 and 5	[26]
QIGLF, TNGIIR, RVPSL, RADHP, YAEER, YPI	Egg white proteins	Antihypertensive; resistant to further gastrointestinal (GI) digestion once formed	Secondary source (review of enzyme, simulated-digestion and animal studies)	Not assigned (secondary source)	[27]
FRADHPFL, YAEERYPIL	Ovalbumin (egg white)	Antihypertensive precursor sequences; further hydrolyzed rather than persisting intact	Secondary source (review of enzyme, simulated-digestion and animal studies)	Not assigned (secondary source)	[27]
Four yolk-hydrolysate peptides: YINQMPQKSRE, YINQMPQKSREA, VTGRFAGHPAAQ, IYEAVNKVSPRAGQF	Apolipoprotein B, vitellogenin-2, apovitellenin-1	Antioxidant, ACE-inhibitory and/or antidiabetic (DPP4 and α-glucosidase inhibition) activities of the synthesized peptides	Chromatographic isolation + mass spectrometry (MS) identification; synthetic peptides tested in biochemical assays	Category 2	[28]
Ovomucin hydrolysates (Protex 26L and pepsin/pancreatin; peptide mixtures not defined at sequence level)	Ovomucin	Anti-adhesive vs. ETEC K88; barrier-protective; anti-inflammatory	Caco-2; IPEC-J2	Category 3	[29]

→ indicates sequential steps of the experimental workflow (prediction, cell assay, in vitro digestion, animal study).

**Table 2:** Major egg-derived bioactive compounds, molecular origin, reported activities, and evidence category.

Compound	Origin	Reported Activity	Evidence Substrate	Highest Evidence Category	Ref.
Ovalbumin	Egg white (~54% of protein)	Storage protein; no firmly established defense role	PP	Not applicable (compositional)	[1]
Ovotransferrin	Egg white (~10%)	Iron restriction (antibacterial); iron-independent membrane-perturbing antibacterial activity	PP	Category 2	[18]
Ovomucoid (Gal d 1)	Egg white (~10%)	Dominant food allergen; exceptional heat/digestion resistance	PP	Category 6/7 (clinical)	[33]
Ovomucin	Egg white (~3%)	Gel-forming; hydrolysates: anti-adhesive vs. ETEC K88, barrier-protective	PP + H	Category 3	[29]
Lysozyme (Gal d 4)	Egg white (~3%)	Peptidoglycan hydrolysis; GALT immunomodulation independent of enzymatic activity	PP	Not assigned (secondary source)	[19]
Avidin	Egg white (minor)	Biotin binding; causes biotin deficiency if raw egg consumed long-term in quantity	PP	Category 6 (single human case report)	[34]
Phosvitin	Yolk granule fraction	Iron chelation, antioxidant; resistant to proteolysis	PP + H	Category 2	[1]
Livetins (α, β, γ/IgY)	Yolk plasma fraction	γ-livetin = IgY (antibody); fraction suppresses LPS-induced macrophage inflammation	PP	Category 3	[31]
Yolk phospholipids (~78.5% phosphatidylcholine)	Yolk (~33% of lipid)	Major dietary choline source; substrate for microbial TMA/TMAO pathway	Comp + Met	Category 7 (choline pathway; Section 6.2)	[11,35]

PP, parent protein; H, hydrolysate preparation; Comp, compositional or structural analysis; Met, metabolite or metabolic pathway observation. Evidence substrate reflects the molecular entity on which the cited evidence is based, not the entity present in whole egg.

**Table 3:** Effects of processing and gastrointestinal digestion on egg-derived bioactives.

Intervention	Compound/Matrix	Reported Effect	Model	Ref.
Cooking vs. raw	Whole egg protein	True ileal digestibility: 90.9% cooked vs. 51.3% raw	Human ileostomy (stable isotope)	[39]
Heat pretreatment, 4–100 °C	Egg white protein	Pepsin digestibility peaks near 65 °C; pepsin + pancreatin digestibility rises continuously to 100 °C; more low-molecular-weight peptides at higher temperature	Simulated digestion	[40]
pH × heat treatment	Egg white powder	Jointly determine protein aggregation/solubility, shaping downstream antioxidant peptide release	Simulated digestion	[41]
Cooking method (hard-boiled, poached, omelet)	Whole egg	Omelet: up to 37% less proteolysis under simulated elderly GI model vs. standard adult; hard-boiled/poached comparatively more digestible	Simulated digestion (age-adjusted)	[42]
Digestion scenario (infant/fed-adult/fasted-adult)	Isolated lysozyme, ovalbumin; food matrices	Infant model leaves proteins substantially intact; adult scenarios achieve more extensive proteolysis; peptides >9 amino acids persist across all scenarios	Simulated digestion (INFOGEST-consistent)	[44]
Cooking + digestion	Omega-3/carotenoid-enriched eggs	Enriched fatty acid bioaccessible regardless of cooking method; lutein bioaccessibility generally decreases post-digestion; peptide content largely unchanged by digestion step	Simulated digestion	[45]

**Table 4:** Mechanisms linking egg-derived bioactives to intestinal, immune, and metabolic responses.

Compound	Model/Challenge	Mechanism Demonstrated	Evidence Category	Ref.
IRW	Caco-2 + LPS	↑TEER, ↓paracellular permeability, restored occludin expression/localization, ↓NF-κB and MAPK activation, direct LPS-neutralizing activity	Category 3	[24]
Ovomucin hydrolysates	Caco-2 + IPEC-J2 + ETEC K88	Restored TEER; sialic-acid-dependent anti-adhesion; ↓NF-κB, ↓MAPK, ↓CaMKII activation, ↓intracellular Ca^2+^, ↓oxidative stress	Category 3	[29]
IRW (not IQW or LKP)	L6 rat myotubes + Ang-II	Improved insulin resistance; corrected oxidative stress response	Category 3	[23]
IRW	SHR rat mesenteric artery	↑ACE2 expression/activity; ↓pro-inflammatory gene expression	Category 5	[22]
IRW	SHR rat (oral)	↓Blood pressure via ACE2/Ang-(1–7)/Mas receptor axis	Category 5	[20]
Lysozyme	Oral administration; GALT/intestinal lymphocytes	Immunomodulation reported as not fully dependent on enzymatic (bacteriolytic) activity	Not assigned (secondary source)	[19]

↑, increase or upregulation; ↓, decrease, reduction or inhibition.

**Table 5:** Primary human studies of egg intake and gut microbiome composition or diversity (the six studies identified in [11], consulted in their original reports).

Study	Design and Population	Egg Exposure (Assessment)	Duration	Microbiome Method	Main Microbiome Findings
Zhu 2020 [13]	Randomized crossover trial; overweight postmenopausal women with mild hypercholesterolemia (n = 20)	2 whole eggs/day vs yolk-free substitute; 4-week washout	4 weeks per period	16S rRNA	No change in microbiome composition; large inter-individual variability
De Filippis 2016 [14]	Cross-sectional; omnivores, vegetarians and vegans, Italy (n = 153)	7-day weighed food diary (egg one of many food variables)	Habitual intake	16S rRNA	No significant association with egg intake; fecal SCFA also not significantly associated
Noh 2021 [15]	Cross-sectional; healthy Korean adults (n = 222)	106-item FFQ (previous year)	Habitual intake	16S rRNA	No significant pattern related to egg intake
Renall 2023 [16]	Cross-sectional; Pacific and New Zealand European women, normal or obese BMI (n = 286)	5-day estimated food record and 220-item FFQ	Habitual intake	Shotgun metagenomics	Higher evenness (Pielou) and butyrate-associated taxa with higher egg intake (*E. rectale*, *F. prausnitzii*, *A. muciniphila*); negative: *B. adolescentis*, *B. bifidum*
Li 2022 [12]	Prospective cohort (Men’s Lifestyle Validation Study); US male health professionals (n = 307)	Repeated FFQs and 7-day diet records	About 6 months of repeated sampling	Shotgun metagenomics	Positive: *B. bifidum* and three other species; negative: two species
Asnicar 2021 [17]	Non-randomized intervention study (PREDICT 1); adults, UK and USA (n = 1098)	Habitual intake (FFQ; tools differed between countries)	14 days (stool at days 0 and 14)	Shotgun metagenomics	Positive: *E. eligens* and two Firmicutes taxa; negative: *B. adolescentis*, *B. catenulatum*, *B. longum* and others

BMI, body mass index; FFQ, food frequency questionnaire; SCFA, short-chain fatty acid. Findings for taxa are taken from [11] and the original reports.

**Table 6:** Microbiome-related effects reported for purified egg-derived peptides and for the phosphatidylcholine–TMAO pathway.

Exposure	Reported Microbiome-Related Effect	Method	Model	Ref.	Key Limitations
IRW (oral)	↑ Shannon index, ↓ Simpson index (fecal) vs. LPS-challenged controls	16S-based diversity indices	Rat (LPS peritonitis)	[50]	Rat model only; egg was gavaged as isolate; not tested from whole dietary egg; no human data
IRW + IQW (oral)	No significant change in overall diversity indices; host serum metabolomic profile altered; infection itself shifted Firmicutes/Actinobacteria down, Bacteroidetes/Proteobacteria up	Microbial + metabolomic profiling	Mouse (C. rodentium infection)	[25]	Mouse model; C. rodentium infection model; limited translatability; short-term only
IRW (oral, DSS colitis)	Alleviated colonic injury; altered abundance of Bacillota and other taxa; changed fecal SCFA levels	Microbiota sequencing; fecal SCFA	Mouse (DSS colitis)	[47]	Mouse model; DSS colitis not directly relevant to healthy humans; no pharmacokinetic data
IRW or IQW (dietary, DSS colitis)	Increased a diversity index and antioxidant enzyme activities; shifted several genera	Microbiota sequencing	Mouse (DSS colitis)	[48]	Observational; egg one of many dietary variables; 16S resolution; no mechanistic data
Two hard-boiled eggs plus a phosphatidylcholine supplement	Antibiotic suppression of the gut microbiota markedly lowered the postprandial TMAO rise, which reappeared after antibiotics were withdrawn	Postprandial plasma and urinary TMAO	Human intervention (healthy participants)	[35]	Observational; FFQ exposure assessment; egg intake confounded with overall diet quality

↑, increase; ↓, decrease.

**Table 7:** Multidimensional evidence matrix for egg-derived bioactives and associated claims.

Compound or Claim	Molecular ID	Digestive Release/Stability	Transport/Bioavailability	Mechanism	Animal Efficacy	Human Exposure/PK	Human Efficacy	Safety	Key Refs.
IRW	Y	P	P	Y	Y	N	N	N	[20,22,23,24,27,46,47,48,50]
IQW	Y	P	N	P	P	N	N	N	[20,23,25,27,48,49]
LKP	Y	P	N	P	N	N	N	N	[20,23,27]
Ovomucin hydrolysates	P	P	N	Y	N	N	N	N	[29]
Lysozyme (HEWL)	Y	n.a.	n.a.	P	P	N	N	P	[19,33]
Yolk peptide AAQEKIRYW	Y	P	N	Y	P	N	N	N	[26]
Cooked vs. raw egg protein digestibility	n.a.	Y	P	P	n.a.	P	n.a.	n.a.	[39,40,42]
Choline→TMA→TMAO after egg intake	Y	n.a.	Y	Y	n.a.	Y	P	P	[11,35]
Whole-egg intake and gut microbiota	n.a.	n.a.	n.a.	N	n.a.	P	P	n.a.	[11,12,13,14,15,16,17]
Egg allergens (Gal d 1–5)	Y	Y	n.a.	Y	n.a.	n.a.	n.a.	Y	[33,60]
Avidin–biotin interaction	Y	P	P	Y	n.a.	n.a.	P	P	[34]

Codes: Y, evidence identified for the dimension; P, partial, indirect, in vitro-only or single-study evidence; N, no study addressing this dimension retrieved by the documented search (Section 1.1)—not a confirmed negative finding; n.a., not applicable. These codes do not constitute a quality hierarchy. Mechanism evidence in cells or animals does not imply human exposure, bioavailability, or clinical efficacy. Codes summarize evidence cited in the sections indicated by the references and do not rank study quality. PK, pharmacokinetics; HEWL, hen egg white lysozyme. →, sequential metabolic conversion (choline to TMA to TMAO).

**Table 8:** Major knowledge gaps and illustrative research approaches.

Knowledge Gap	Illustrative Research Approach	Timeframe	Current Evidence Basis
Unknown whether IRW/IQW/LKP are generated/survive digestion of whole cooked egg	Targeted liquid chromatography–tandem mass spectrometry (LC-MS/MS) peptidomic screening of INFOGEST whole-egg digests across physiological scenarios	Short-term	Preclinical/in vitro only
Purified-peptide findings untested against real digests	Re-run Caco-2/IPEC-J2 barrier assays using whole-egg digestate in place of purified peptide	Short-term	Preclinical/in vitro only
Hen source variables unreported across studies	Reporting standard for breed, diet and husbandry	Short-term	None retrieved
No human data on IRW/IQW/LKP absorption	Human pharmacokinetic study, targeted LC-MS/MS, postprandial time course	Medium-term	None retrieved
Short trial duration vs. long observational associations	Intervention of several months (for example, 6–12 months) with shotgun metagenomic sampling at multiple time points	Medium-term	Human (observational only)
Compositional microbiome data without functional confirmation	Paired metatranscriptomic or metabolomic sampling alongside taxonomic sequencing	Medium-term	Human (observational only)
No dose–response relationship established	Graded whole-egg intake (for example, 0/1/2/4 eggs/day, the range used in prior trials [11]) with standardized hen source	Medium-term	Human (observational; short RCT)
No dedicated peptide safety/toxicology data	Independent dose-escalation toxicological evaluation, including determination of the no-observed-adverse-effect level	Medium-term	None retrieved
Precision nutrition untested as a predictive (not just descriptive) framework	Long-duration intervention stratified by FMO3 genotype, renal function and, if justified, baseline microbiome	Long-term	None retrieved
No standardized egg-specific bioaccessibility/bioavailability reporting	Consensus reporting method built on, and extending, INFOGEST	Long-term	Preclinical/in vitro only
Habitual-intake microbiome associations remain confounded	Prospective cohort with repeated dietary and shotgun metagenomic measures; FMO3 genotype as stratification variable, not an instrument for egg intake	Long-term	Human (observational only)

The approaches in Table 8 are examples of how each gap could be addressed. They are not preferred protocols, and doses, durations and sample sizes would require justification by pilot data and power calculations.

## Data Availability

No new data were created or analyzed in this study. Data sharing is not applicable to this article.

## Supplementary Materials

The following supporting information can be downloaded at: https://www.mdpi.com/article/10.3390/foods15213849/s1, Table S1: literature search strategies and numbers of records by database.

## Abbreviations

The following abbreviations are used in this manuscript:

Abbreviation	Definition
ACE	Angiotensin-converting enzyme
CaMKII	Calcium/calmodulin-dependent protein kinase II
ESM	Eggshell membrane
ETEC	Enterotoxigenic *Escherichia coli*
FMO3	Flavin-containing monooxygenase 3
GALT	Gut-associated lymphoid tissue
HEWL	Hen egg white lysozyme
IgY	Immunoglobulin Y
LPS	Lipopolysaccharide
MAPK	Mitogen-activated protein kinase
NF-κB	Nuclear factor kappa-light-chain-enhancer of activated B cells
SCFA	Short-chain fatty acid(s)
SHR	Spontaneously hypertensive rat(s)
TEER	Transepithelial electrical resistance
TLR	Toll-like receptor
TMA	Trimethylamine
ACE2	Angiotensin-converting enzyme 2
AMPK	AMP-activated protein kinase
AUC	Area under the curve
CKD	Chronic kidney disease
CRP	C-reactive protein
DPP4	Dipeptidyl peptidase-4
DSS	Dextran sulfate sodium
FFQ	Food frequency questionnaire
IL-6	Interleukin-6
IPEC-J2	Intestinal porcine epithelial cell line J2
IRW, IQW, LKP	Ile-Arg-Trp, Ile-Gln-Trp, Leu-Lys-Pro
LC-MS/MS	Liquid chromatography–tandem mass spectrometry
MALDI-ToF	Matrix-assisted laser desorption/ionization time-of-flight
MS	Mass spectrometry
Nrf2	Nuclear factor erythroid 2-related factor 2
PK	Pharmacokinetics
RCT	Randomized controlled trial
SHIME	Simulator of the Human Intestinal Microbial Ecosystem
TIM	TNO gastrointestinal model
TNF-α	Tumor necrosis factor-α
TMAO	Trimethylamine-N-oxide
