# Supplementary material for: Hen Egg-Derived Bioactive Proteins and Peptides at the Gut Interface: From Molecular Characterization and Digestion to Microbiome Interactions and Prospective Precision Nutrition
Source: Foods. 2026 Oct 28;15(21):3849. doi: 10.3390/foods15213849 (PMC13762729; doi:10.3390/foods15213849)
Supplement: Supplementary file 1 [file foods-15-03849-s001.zip › foods-4562659-supplementary.pdf]

Table S1. Literature Search Strategies and Record Counts

Manuscript: foods-4562659 | Journal: Foods (MDPI)

Searches conducted: 13 October 2026 | Databases: PubMed/MEDLINE and Europe PMC

This table provides the full search strings, date of search, and record counts for the two-database structured search underpinning this narrative review, as referenced in Section 1.1. The search was not a systematic review search and was not registered. Scopus and Web of Science were not available to the authors and were not searched; this is acknowledged as a limitation throughout the review.

Table S1a. PubMed/MEDLINE Search Strings by Concept Block

All searches: titles and abstracts [tiab]; language: English; date range: database inception to 13 October 2026. Each block was combined independently with the egg-terms string.

Block	Search string (PubMed/MEDLINE; titles & abstracts)	Scope / Concept	Records
Block A — Peptides & Bioactivity	("egg"[tiab] OR "egg white"[tiab] OR "egg yolk"[tiab] OR ovotransferrin[tiab] OR ovomucin[tiab] OR lysozyme[tiab] OR phosvitin[tiab] OR livetin[tiab] OR "eggshell membrane"[tiab]) AND ("bioactive peptide"[tiab] OR hydrolysate[tiab] OR IRW[tiab] OR IQW[tiab] OR LKP[tiab])	Peptide identification, ACE inhibition, antihypertensive, anti-inflammatory peptides	596
Block B — Digestion & Bioaccessibility	("egg"[tiab] OR "egg white"[tiab] OR "egg yolk"[tiab] OR ovotransferrin[tiab] OR ovomucin[tiab] OR	Simulated GI digestion, INFOGEST protocol,	812

مقيد - Restricted

	lysozyme[tiab] OR phosvitin[tiab] OR livetin[tiab] OR "eggshell membrane"[tiab]) AND ("in vitro digestion"[tiab] OR INFOGEST[tiab] OR bioaccessib*[tiab] OR bioavailab*[tiab])	bioaccessibility, bioavailability	
Block C — Intestinal Barrier	("egg"[tiab] OR "egg white"[tiab] OR "egg yolk"[tiab] OR ovotransferrin[tiab] OR ovomucin[tiab] OR lysozyme[tiab] OR phosvitin[tiab] OR livetin[tiab] OR "eggshell membrane"[tiab]) AND ("intestinal barrier"[tiab] OR Caco-2[tiab] OR "tight junction"[tiab])	Tight junction proteins, TEER, Caco-2 permeability, epithelial barrier function	554
Block D — Gut Microbiome & Metabolites	("egg"[tiab] OR "egg white"[tiab] OR "egg yolk"[tiab] OR ovotransferrin[tiab] OR ovomucin[tiab] OR lysozyme[tiab] OR phosvitin[tiab] OR livetin[tiab] OR "eggshell membrane"[tiab]) AND ("gut microbio*"[tiab] OR metagenom*[tiab] OR "16S"[tiab] OR TMAO[tiab] OR choline[tiab])	Gut microbiota composition, TMAO, choline metabolism, metagenomics, 16S rRNA	2,046
Block E — Precision Nutrition	("egg"[tiab] OR "egg white"[tiab] OR "egg yolk"[tiab] OR ovotransferrin[tiab] OR ovomucin[tiab] OR lysozyme[tiab] OR	Personalized / precision nutrition frameworks, individual	29

مقيد - Restricted

	phosvitin[tiab] OR livetin[tiab] OR "eggshell membrane"[tiab]) AND ("precision nutrition"[tiab] OR "personalized nutrition"[tiab] OR "interindividual variab*"[tiab])	response variation	
Total (PubMed, before deduplication)			4,037

Egg-terms string (used in all 5 blocks): ("egg"[tiab] OR "egg white"[tiab] OR "egg yolk"[tiab] OR ovotransferrin[tiab] OR ovomucin[tiab] OR lysozyme[tiab] OR phosvitin[tiab] OR livetin[tiab] OR "eggshell membrane"[tiab])

Table S1b. Europe PMC Search String

A single combined search was run in Europe PMC using all concept blocks joined by OR, combined with the egg-terms string.

Database	Search string (titles & abstracts)	Date	Records
Europe PMC	(egg OR "egg white" OR "egg yolk" OR ovotransferrin OR ovomucin OR lysozyme OR phosvitin OR livetin OR "eggshell membrane") AND ("bioactive peptide" OR hydrolysate OR IRW OR IQW OR LKP OR "in vitro digestion" OR INFOGEST OR bioaccessib* OR bioavailab* OR "intestinal barrier" OR Caco-2 OR "tight junction" OR "gut microbio*" OR metagenom* OR TMAO OR choline OR "precision nutrition" OR "personalized nutrition") [TITLE_ABS]	13 Oct 2026	3,538

Table S1c. Record Flow Summary

As this is a narrative review, a full PRISMA flow diagram was not produced. The table below summarises the approximate flow of records from retrieval to inclusion.

مقيد - Restricted

Stage	Count	Note
PubMed/MEDLINE — total across 5 blocks	4,037	Block A: 596; Block B: 812; Block C: 554; Block D: 2,046; Block E: 29
Europe PMC — combined search	3,538	Single combined search across all concept blocks
Combined total (pre-deduplication)	7,575	Sum of PubMed + Europe PMC totals
Estimated duplicates removed	~1,200–1,500	Title and first-author matching; manual review (no deduplication software used)
Records after deduplication (estimated)	~6,000–6,300	Narrative review — exact count not tracked at this stage
Records excluded at title/abstract screening	Majority	Outside defined scope (see Section 1.1 inclusion/exclusion criteria)
Records retrieved for full-text assessment	Not formally counted	Narrative design — formal PRISMA flow not applicable
Studies contributing primary data to review	Approx. 60+	As reflected in reference list (primary studies and systematic reviews combined)

Search Limitations

1. Only two databases were searched (PubMed/MEDLINE and Europe PMC). Scopus and Web of Science were not available and were not searched. Studies indexed only in those databases may not have been retrieved.

2. The search was limited to English-language records. Relevant studies published in other languages may have been missed.

3. No deduplication software was used. Duplicate removal was performed manually by title and first-author matching; exact counts at the deduplication and screening stages were not formally recorded, as the review was not designed as a systematic review.

4. All statements in the manuscript that "no study was identified" refer specifically to this two-database search conducted to 13 October 2026 and should not be interpreted as confirmed global absence of evidence.

5. Grey literature, conference abstracts and pre-prints were excluded.

مقيد - Restricted
